# Wild-type p53 upregulates an early onset breast cancer-associated gene GAS7 to suppress metastasis via GAS7–CYFIP1-mediated signaling pathway

**DOI:** 10.1038/s41388-018-0253-9

**Published:** 2018-04-30

**Authors:** Jer-Wei Chang, Wen-Hung Kuo, Chiao-Mei Lin, Wen-Ling Chen, Shih-Hsuan Chan, Meng-Fan Chiu, I-Shou Chang, Shih-Sheng Jiang, Fang-Yu Tsai, Chung-Hsing Chen, Pei-Hsin Huang, King-Jen Chang, Kai-Ti Lin, Sheng-Chieh Lin, Ming-Yang Wang, Yih-Huei Uen, Chi-Wen Tu, Ming-Feng Hou, Shih-Feng Tsai, Chen-Yang Shen, Shiao-Lin Tung, Lu-Hai Wang

**Affiliations:** 1 Institute of Molecular and Genomic Medicine, National Health Research Institute, Miaoli, Taiwan; 2 Department of Surgery, National Taiwan University Hospital, Taipei, Taiwan; 3 Institute of Molecular Medicine, College of Life Science, National Tsing Hua University, Hsinchu, Taiwan; 4 Graduate Institute of Integrated Medicine, College of Chinese Medicine, China Medical University, Taichung, Taiwan; 5 National Institute of Cancer Research, National Health Research Institutes, Miaoli, Taiwan; 6 Department of Pathology, College of Medicine, National Taiwan University, Taipei, Taiwan; 7 Taiwan Adventist Hospital, Taipei, Taiwan; 8 Institute of Biotechnology, College of Life Science, National Tsing Hua University, Hsinchu, Taiwan; 9 College of Chinese Medicine, China Medical University, Taichung, Taiwan; 10 Department of Surgery, Asia University Hospital, Taichung, Taiwan; 11 Department of General Surgery, Ditmanson Medical Foundation Chia-Yi Christian Hospital, Chiayi, Taiwan; 12 Department of Surgery, College of Medicine, Kaohsiung Medical University, Kaohsiung, Taiwan; 13 Department of Surgery, Kaohsiung Municipal Hsiao Kang Hospital, Kaohsiung, Taiwan; 14 Division of Breast Surgery, Kaohsiung Medical University Hospital, Kaohsiung, Taiwan; 15 Institute of Biomedical Sciences, Academia Sinica, Taipei, Taiwan; 16 Department of Hematology and Oncology, Ton-Yen General Hospital, Hsinchu, Taiwan

## Abstract

The early onset breast cancer patients (age ≤ 40) often display higher incidence of axillary lymph node metastasis, and poorer five-year survival than the late-onset patients. To identify the genes and molecules associated with poor prognosis of early onset breast cancer, we examined gene expression profiles from paired breast normal/tumor tissues, and coupled with Gene Ontology and public data base analysis. Our data showed that the expression of *GAS7b* gene was lower in the early onset breast cancer patients as compared to the elder patients. We found that GAS7 was associated with CYFIP1 and WAVE2 complex to suppress breast cancer metastasis via blocking CYFIP1 and Rac1 protein interaction, actin polymerization, and β1-integrin/FAK/Src signaling. We further demonstrated that p53 directly regulated *GAS7* gene expression, which was inversely correlated with p53 mutations in breast cancer specimens. Our study uncover a novel regulatory mechanism of p53 in early onset breast cancer progression through GAS7–CYFIP1-mediated signaling pathways.

## Introduction

In the United States, around 6.6% of breast cancer cases are diagnosed below age of 40 years old [1], whereas in Taiwan, that is about 29.3%. The peak initial diagnosis age of breast cancer among Taiwanese women is about 10–15 years younger than that in Caucasian Americans [2, 3]. The breast cancer diagnosed with age less than 40 years old are more aggressive, and five-year survival rate of those patients is poorer than the late-onset patients [2, 4–6]. Furthermore, early onset patients often display higher incidence of axillary lymph node metastasis [2], and primary breast tumor with earlier onset time is more likely to develop bilateral breast cancer [7]. Those findings imply that tumor cells in early onset breast cancer patients possess higher metastatic characteristics. Identification of molecules and signaling pathways regulating early onset breast cancer would be instrumental for prognosis and development of treatment strategies.

We performed exon array assays and to align with public domain database to identify potential gene(s) associated with early onset breast cancer. We found that the expression level of growth-arrest-specific 7 isoform b (*GAS7b*) gene was lower in early onset compared to the late onset breast cancer cells. Previous studies have demonstrated that GAS7 can promote neurite-like outgrowth of cells, and it co-localizes with microfilaments in membrane ruffles concurrent with actin assembly and membrane outgrowth [8, 9]. These findings suggest that GAS7 may play a role in regulating cell structure and migration. However, the role of GAS7 in cancer has not yet been discussed.

The WisKott–Aldrich syndrome protein (WASP) and WASP-family verprolin-homologous (WAVE) family proteins are known to regulate actin polymerization during formation of filopodia and lamellipodia in cell migration [10, 11]. The GTP-bound active form of Rac1 activates WAVE2 complex through direct interaction with CYFIP1/SRA1, a subunit of the WAVE2 protein complex, to stimulate Arp2/3-mediated actin polymerization [10, 12]. In this study, we demonstrated that GAS7b interacted with CYFIP1 protein, and disrupted interaction between CYFIP1 and active form of Rac1, leading to blocking of actin polymerization, and reduced β1-integrin/FAK/Src signaling. This resulted in the suppression of breast cancer cell migration/invasion and metastasis. We also showed that wild-type, but not mutant, p53 could bind to *GAS7* gene promoter and to promote its transcription. As a result, a decreased *GAS7b* expression is associated with p53 gene mutations, which occur at a higher rate in early onset breast cancer patients. Clinical data and public domain data sets reveal that reduced *GAS7* expression is associated with lymph-node metastasis and poor overall survival. In conclusion, our study identified *GAS7b* to be related to poor prognosis of early onset breast cancer and can serve as a potential prognostic biomarker for breast cancer metastasis.

## Result

### Lower *GAS7b* expression in breast tumor tissue correlates with early onset breast cancer

To investigate the genes involved in poor prognosis of early onset breast cancer, we performed exon array assays and aligned with public domain database to identify potential gene(s) associated with early onset breast cancer. The detailed analytic processes are shown in Supplementary Figures S1 and S2. The *GAS7* gene showed significantly lower expression in the early onset breast cancer patients (≤40 years old) than in elder patients (Supplementary Figure S1). Therefore, we decided to further investigate the role of GAS7 in early onset breast cancer.

**Fig. 1:**
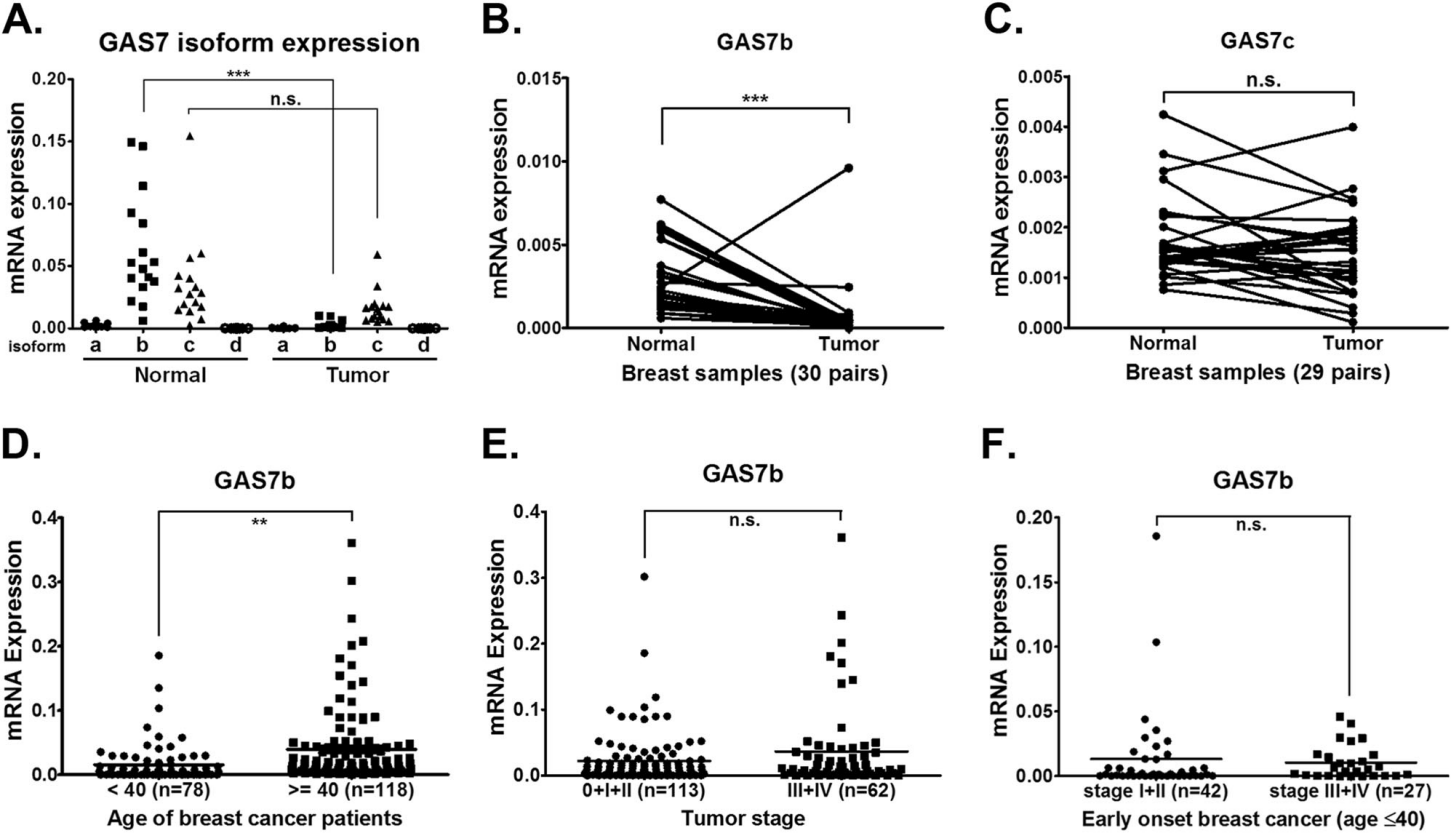
*GAS7b* mRNA expression level is lower in early onset breast cancer samples. **a** The mRNA expression levels of four isoforms (*GAS7a*, *GAS7b*, *GAS7c*, and *GAS7d*) of *GAS7* in 16 pairs of breast normal/tumor tissue specimens were analyzed by RT-qPCR. **b** *GAS7b* mRNA expression levels in 30 pairs of breast normal/tumor tissue specimens were analyzed by paired *t*-test. **c** *GAS7c* mRNA expression levels in 29 pairs of breast normal/tumor tissue specimens were analyzed by paired *t*-test. **d** *GAS7b* mRNA expression levels between early onset breast cancer patients (age ≤ 40 years old, *n* = 78) and older patients (age > 40 years old, *n* = 118) were analyzed. **e** *GAS7b* mRNA expression levels for tumors of earlier (stages 0+I+II, *n* = 113) versus latter stages (stages III+IV, *n* = 62) were analyzed. **f** *GAS7b* mRNA expression levels for early (*n* = 42) and late (*n* = 27) tumor stages within the ≤40 year-old age group were analyzed. Two-tailed *t*-test was used for statistical analysis (**p < 0.01; ***p < 0.001; n.s. non-significant)

*GAS7* gene encodes four protein isoforms via alternative splicing. To study the expression of different *GAS7* isoforms in breast cancer patients, quantitative RT-PCR was conducted from 16 pairs of breast normal/tumor tissue specimens. The results showed that both isoform *GAS7b* and *GAS7c* were expressed in normal and tumor breast tissues, but *GAS7b* expression was significantly lower in tumors (Fig. 1a). We subsequently collected 30 more pairs of breast normal/tumor tissue specimens, and found that mRNA levels of *GAS7b* were significantly lower in tumor tissues than those in normal tissues, but this phenomenon was not found for *GAS7c* mRNA (Fig. 1b, c). We then further quantified *GAS7b* mRNA levels from another 196 breast tumor samples (175 samples with tumor stage information), and observed a significantly downregulated *GAS7b* expression in the early onset breast cancer patients (≤40 years old) as compared to the late onset patients (Fig. 1d). However, there were no significant differences in *GAS7b* expression between early stages (stages 0+I+II) and late stages (stages III+IV) tumors (Fig. 1e), nor between the early and late stage tumors from patients less than 40 years old (Fig. 1f). These data suggested that lower expression of *GAS7b* appeared to be associated with early onset breast cancer, but not with its clinical stages.

### Overexpression of GAS7b decreases breast cancer cell proliferation, migration, invasion, and adhesion **in vitro**

To explore the role of GAS7b in breast cancer cells, various functional assays were performed. Immunoblotting results indicated that GAS7b expression in most of breast cancer cell lines was low, with the exception of MCF-7 cells (Supplementary Figure S3A). The correlation of proliferation rate with GAS7b expression levels in MDA-MB-231 and MCF-7 breast cancer cells was examined. By performing MTS assays, we found that overexpressing GAS7b in MDA-MB-231 cells reduced their proliferation (Supplementary Figure S3B). Conversely, knockdown of GAS7 in MCF-7 cells increased their proliferation (Supplementary Figure S3C). Moreover, we found increased p21 and p27 cell cycle regulator proteins expression in MDA-MB-231 cells overexpressing GAS7b (Supplementary Figure S3D). These observations suggested that GAS7 was involved in cell cycle regulation.

**Fig. 2:**
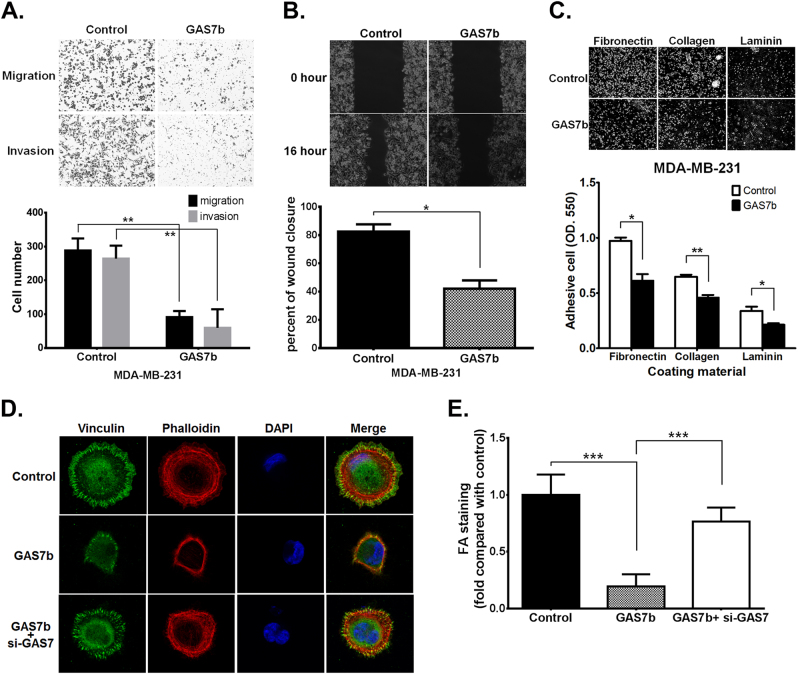
GAS7b inhibits MDA-MB-231 breast cancer cell migration, invasion, cell adhesion, and FA formation. MDA-MB-231 cells were transfected with control or GAS7b-expressing plasmids. After 48 h, the cells were subjected to the following assays. **a** Upper: representative photographs of the trans-well cell migration and invasion assays. Bottom: statistical analysis of migrated and invaded cells are shown by histograms. Data represent normalized mean ± SD (*n* = 3). **b** Upper: representative photographs of wound healing cell migration assay. Bottom: statistical analysis of cell migrated areas are shown by histograms. Data represent normalized mean ± SD (*n* = 3). **c** Upper: representative photographs of the cell adhesion assay using fibronectin, type I collagen, or laminin coated plates. Bottom: the adhesion cells were stained with crystal violet, and the eluted dye was measured by spectrophotometer. Data represent normalized mean ± SD (*n* = 3). **d** Immunofluorescence staining and confocal microscopy of Vinculin (green) for FA, Phalloidin (red) for F-actin, and DAPI (blue) for nuclear staining. MDA-MB-231 cells were transfected with control vector, GAS7b-expressing plasmid, or GAS7b-expressing plasmid plus GAS7 siRNA, and cells were plated on fibronectin coated coverslips for 90 min, and **e** quantification of total focal adhesions per cell (*n* = 10, mean ± SD). Two-tailed *t*-test was used for these statistical analysis (*p < 0.05; **p < 0.01; ***p < 0.001)

Previous study has shown that GAS7b mediated actin polymerization and promoted neurite-like outgrowth of neurons [8]. We therefore investigated the role of GAS7b in cancer cell migration/invasion in vitro. Overexpression of GAS7b in MDA-MB-231 cells significantly inhibited cell migration and invasion in trans-well assays (Fig. 2a), as well as migration in wound healing assays (Fig. 2b). We further studied the inhibitory mechanism of GAS7b in MDA-MB-231-IV2 cells, which process higher ability in migration and invasion than the parental MDA-MB-231 cells [13]. The results showed that overexpression of GAS7b affected the cell shape and inhibited the migratory ability of MDA-MB-231-IV2 cells (Supplementary Figure S4A and S4B). Time-lapse analysis of cell migration showed significant inhibition of total distance traveled upon overexpression of GAS7b in MDA-MB-231-IV2 cells (Supplementary Figure S4C). These results demonstrated that GAS7b affected cell morphology and inhibited cancer cell motility.

The formation of adhesion complexes between cells and their surrounding matrix plays an important role in cell migration. Thus, MDA-MB-231 cells with or without overexpression of GAS7b were seeded onto fibronectin (FN), Type I collagen or laminin-coated plates for the assay of focal adhesions. The result showed that cell adhesion in three different materials were significantly inhibited by overexpression of GAS7b in MDA-MB-231 cells (Fig. 2c), and similar results were also observed in MDA-MB-231-IV2 cells (Supplementary Figure S4D). The effect of GAS7b on the formation of focal adhesions (FAs) in MDA-MB-231 and MDA-MB-231-IV2 cells were further examined by immunofluorescence staining. As shown in Fig. 2d, e and Supplementary Figure S5, overexpression of GAS7b disrupted the spreading efficiency of cells during early phase of seeding, and significantly reduced number and intensity of FAs. This phenomenon was reversed by knockdown of GAS7. The results suggested that GAS7b inhibited breast cancer cells motility as well as cell adhesion, through disruption of FAs formation and the spreading of cells.

### GAS7b is located at cell periphery and interacts with CYFIP1 to suppress the binding of GTP-Rac1

**Fig. 3:**
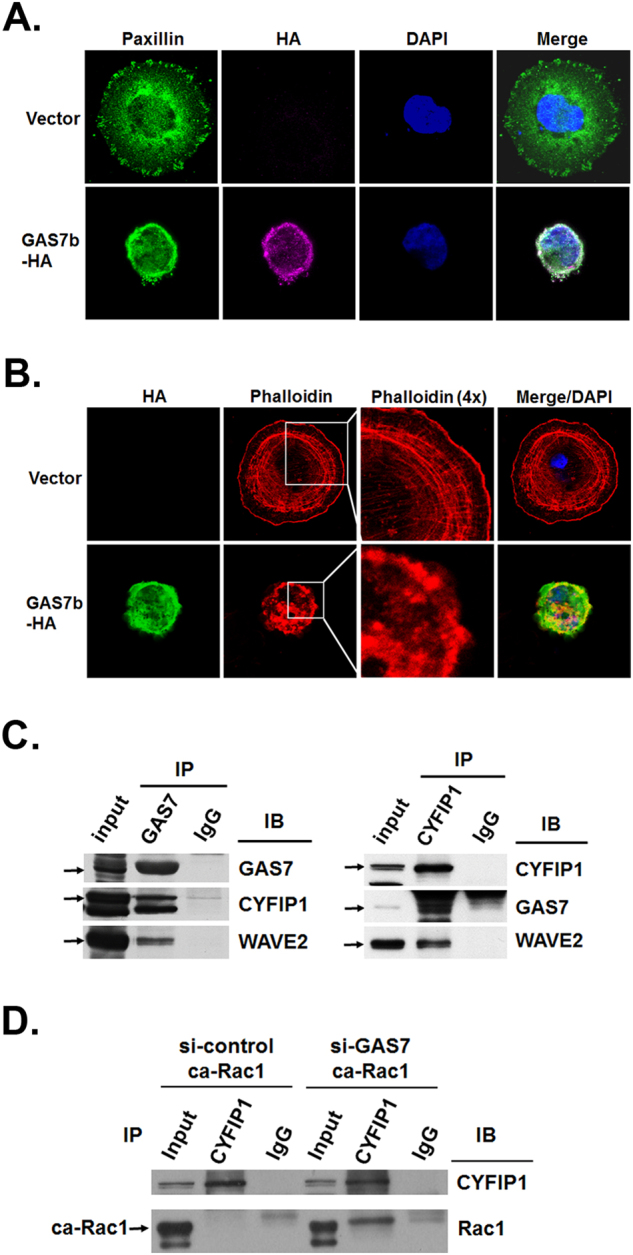
The GAS7b is localized in cell periphery and interacts with CYFIP1 to suppress the binding of Rac1 active form. **a** MDA-MB-231 cells were transfected with HA-tagged-GAS7b expressing plasmid. The cells were plated on fibronectin coated coverslips for 90 min, and confocal microscopy images of Paxillin (green) for FA, HA (purple) for GAS7b, and DAPI (blue) for nuclear staining were taken. **b** The MDA-MB-231 cells expressing HA-tagged-GAS7b were stained with anti-HA (green) antibody, Phalloidin (red), and DAPI (blue), and images from confocal microscopy were taken. The Phalloidin staining for F-actin that zoom in 4× are also shown. **c** Co-immunoprecipitation (co-IP) assay with MCF-7 cells was carried out. Anti-GAS7 and anti-CYFIP1 antibodies were used for IP and western blotting. Normal goat IgG was used as the negative control for IP experiments. **d** MCF-7 cells were transfected with control or GAS7 siRNA, and subsequently super-transfected with the constitutively active Rac1 (ca-Rac1) plasmid. Anti-CYFIP1 was used in the IP assay to pulldown associated proteins, anti-CYFIP1 and anti-Rac1 antibodies were used for western blotting. Normal rabbit IgG was used as the negative control

To further investigate the role of GAS7b in the formation of FAs, we performed immunofluorescence staining to localize the subcellular distribution of GAS7b. The confocal microscopic images showed that GAS7b and Paxillin were co-located in cell periphery (Fig. 3a), suggesting that GAS7b plays a significant role in the cell–matrix interaction and formation of FAs. GAS7b greatly inhibited spreading of MDA-MB-231 cells (Fig. 2d), thus the formation of actin cable was examined. We found that GAS7b did not affect the abundance of actin in MDA-MB-231 cells, but delayed actin polymerization and filaments formation at 1.5 h after seeding (Fig. 3b), and the ability of adhesion was gradually regained after 12 h of seeding (Supplementary Figure S6). This result is in agreement with the previous study in NIH3T3 mouse fibroblasts [9], and suggests that GAS7 functions as a regulator for actin polymerization in breast cancer cells.

A previous study used mass spectrometry to predict molecules that interact with WW domain containing proteins, including GAS7 [14]. Several proteins were found to interact with GAS7, including CYFIP1/SRA1, a subunit of the WASP-family verprolin-homologous protein (WAVE) complex. To assess whether GAS7 was associated with the CYFIP1 protein, we performed the co-immunoprecipitation (co-IP) and western blot analysis with MCF-7 cells. As shown in Fig. 3c, endogenous GAS7 protein could co-precipitate with CYFIP1 and WAVE2 proteins, and reciprocal co-IP also demonstrated that CYFIP1 interacted with GAS7 and WAVE2. To examine co-localization of GAS7b and CYFIP1 protein, MDA-MB-231 cells were transfected with GAS7b-HA expressing plasmid. The result of immunofluorescence staining indicated that GAS7b and CYFIP1 were co-located at the cell periphery (Supplementary Figure S7), and together may play a role in FA formation and regulate cell migration and invasion.

CYFIP1 protein was shown to bind to Rac1 small GTPase, and transmit upstream signals to activate the WAVE2 complex for actin polymerization [10, 12]. To further study the functional relationship between GAS7, CYFIP1, and Rac1 proteins, a co-IP and western blot analysis was performed. The MCF-7 cells were transfected with control or GAS7 siRNA which was designed to target the 3′-UTR of *GAS7* gene to knockdown the endogenous *GAS7* but not the exogenous GAS7b-HA, and the cells were sequentially transfected with empty or GAS7b-HA expression vectors. The result indicated that endogenous Rac1 protein could co-precipitate with CYFIP1 protein upon knockdown of GAS7, but the protein–protein interaction between Rac1 and CYFIP1 was reduced upon GAS7b-HA overexpression (Supplementary Figure S8). Moreover, to investigate whether GAS7 affects the binding of CYFIP1 to the active form of Rac1 (Rac1-GTP), the MCF-7 cells were transfected with control or GAS7 siRNA, and subsequently transfected with constitutively active Rac1 (ca-Rac1) plasmid. The result indicated that ca-Rac1 protein could co-precipitate with the endogenous CYFIP1 protein upon knockdown of GAS7 (Fig. 3d), suggesting GAS7 protein interfered with the protein–protein interaction between CYFIP1 and Rac1-GTP. Overall, these results suggest that GAS7 associates with CYFIP1 to perturb the binding of CYFIP1 and active form of Rac1, thus resulting in the inhibition of actin polymerization in breast cancer cells.

### The GAS7b–CYFIP1 protein complex suppresses breast cancer cell migration and invasion through inhibiting integrin/FAK/Src/Rac1 signaling

**Fig. 4:**
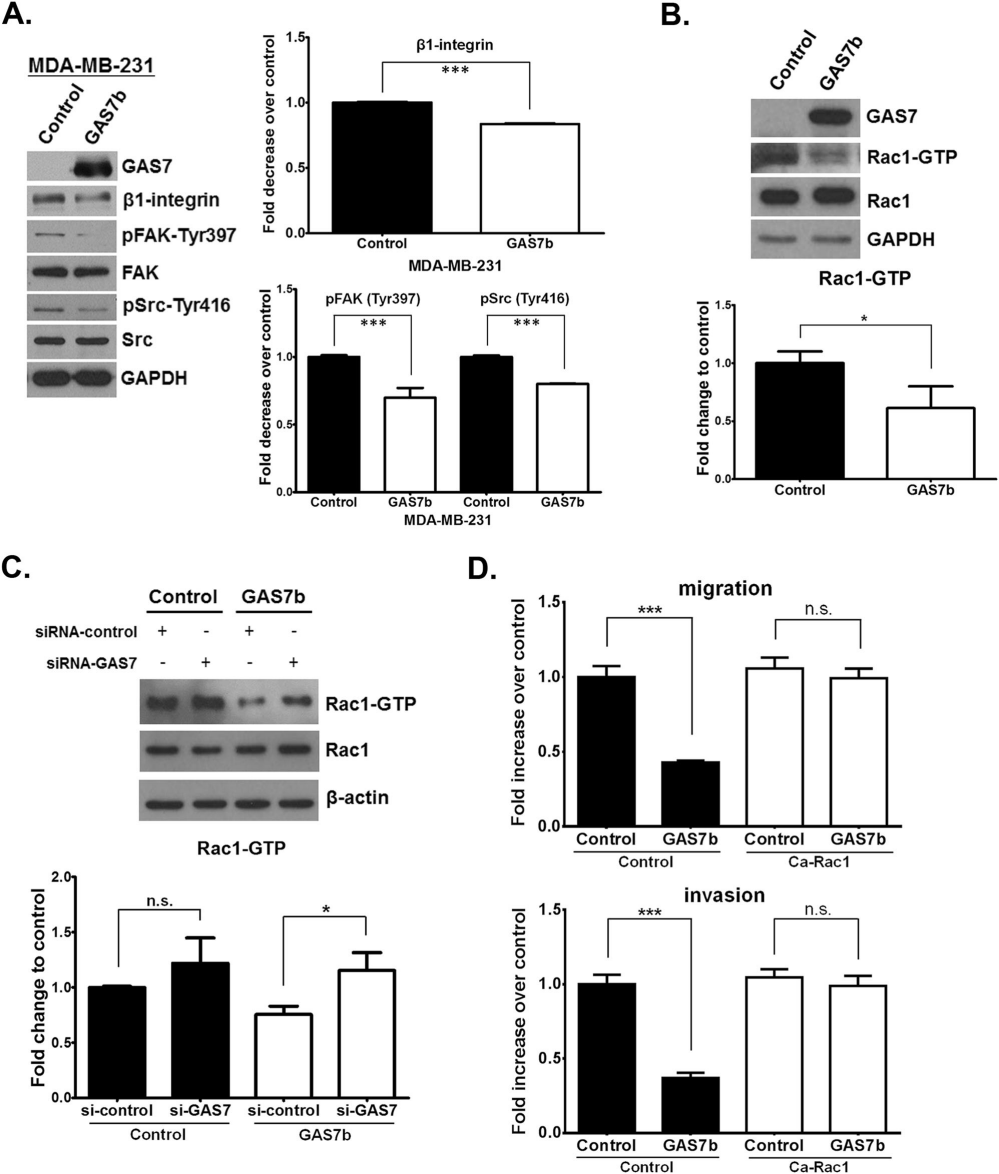
GAS7b suppresses Integrin/FAK/Src/Rac1 signaling of breast cancer cells. **a** Left: Western blotting analysis shows expression of β1-integrin, phospho-FAK (pFAK-Tyr397), FAK, phospho-Src (pSrc-Tyr416), and Src in MDA-MB-231 cells that overexpressing GAS7b. Right: The quantification of β1-integrin, pFAK (Tyr397), and pSrc (Tyr416) expression levels from western blotting analyses. Histograms represent normalized mean ± SD (*n* = 3). **b** Upper: The GST-PAK pull-down analysis to assess the level of Rac1-GTP form in MDA-MB-231 cells with overexpression of GAS7 or control vector. The anti-Rac1 antibody was used for western blotting analysis. Bottom: the levels of Rac1-GTP were quantified and normalized to total Rac1. Histograms represent normalized mean ± SD (*n* = 3). **c** Upper: Western blotting analysis of GST-PAK pull-down assay as described in **b** was performed with MDA-MB-231 cells transfected with control or GAS7-expression vectors, and subsequently transfected with control or GAS7 siRNA. Bottom: the levels of Rac1-GTP were quantified and normalized to total Rac1. Histograms represent normalized mean ± SD (*n* = 3). **d** MDA-MB-231 cells overexpressing GAS7 or control vector, and subsequently transfected with ca-Rac1 or control vector were analyzed. The different transfected cells were examined by trans-well for cell migration (upper) and invasion (bottom) ability. Histograms represent normalized mean ± SD (*n* = 3). Two-tailed *t*-test was used for these statistical analysis (*p < 0.05; **p < 0.01; ***p < 0.001; n.s. non-significant)

Based on our data (Fig. 3), GAS7b caused cell shrinkage, reduced cell adhesion, and hampered cell migration. We suspected that reduced cell–matrix interaction most likely would interfere with integrin-mediated signaling. To test this hypothesis, we examined the major players in the integrin signaling pathway. The results showed that β1-integrin protein was significantly downregulated in the GAS7b overexpressing MDA-MB-231 cells, and so was the phosphorylation of FAK Tyr-397 and Src Tyr-416 (Fig. 4a). These data indicated that overexpression of GAS7b in MDA-MB-231 cells, resulted in reduced β1-integrin protein expression, and inhibition of the FAK–Src signaling. Whereas an increased β1-Integrin protein expression and phosphorylation of FAK Tyr-397 and Src Tyr-416 were observed upon knockdown of GAS7 in MCF-7 cells (Supplementary Figure S9). Our data suggested that GAS7b suppressed breast cancer cell migration through downregulating FAK–Src signaling pathway.

The Rho GTPases family proteins transduce intracellular signals known to regulate a variety of cellular functions, especially through the WAVE complex to modulate cytoskeletal dynamics [15]. We therefore analyzed the relationships between GAS7b and Rac1/Cdc42, which are among the best characterized Rho family members. The Rac1/Cdc42 activation assay revealed that overexpression of GAS7b resulted in decreased level of Rac1-GTP in MDA-MB-231 cells (Fig. 4b), but Cdc42-GTP level was not affected (data not shown). This de-activation was abrogated upon co-transfection with GAS7 siRNA (Fig. 4c). To further confirm that GAS7b suppressed breast cancer cell migration and invasion via inhibition of Rac1 activity, ca-Rac1 plasmid was transfected into MDA-MB-231 cells that overexpressing GAS7b. Indeed, overexpressing ca-Rac1 significantly reversed GAS7b-mediated inhibition of cell migration and invasion (Fig. 4d). Overall, our results demonstrated that GAS7b suppressed breast cancer cells migration and invasion through inhibition of β1-integrin–FAK–Src signaling pathway, and the downstream Rac1 activity.

### Overexpression of GAS7b decreases breast cancer cell growth and metastasis in mouse model

**Fig. 5:**
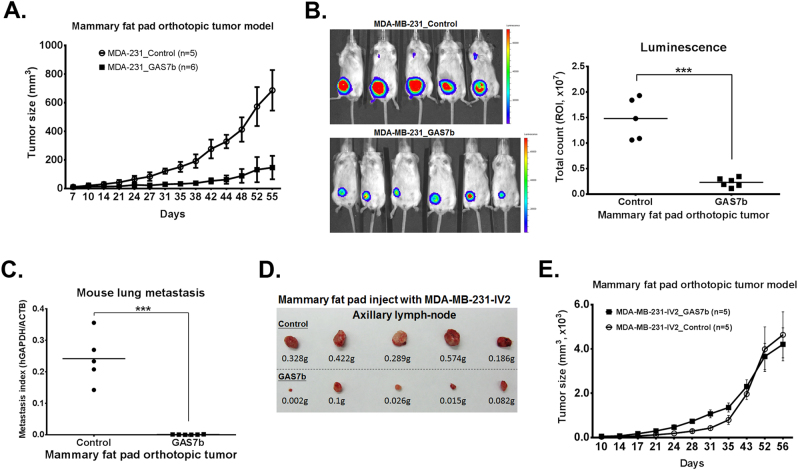
GAS7b suppresses breast cancer cell growth and metastasis in xenograft mouse model. **a** The MDA-MB-231 cells tagged with luciferase protein were stably transfected with GAS7b or empty vector, and the cells (1 × 10^6^ per mouse) were orthotopically implanted into mammary fat pad of SCID mice (control group mice: *n* = 5, GAS7b group mice: *n* = 6). The tumor size was measured twice a week until day 55 post implantation, and the growth curves of primary tumors from the cells overexpressing GAS7b or control vector are shown. Linear regression model was used for statistical analysis, and the data showed statistically significant differences (*p* < 0.001). **b** Left: the primary tumor growth and metastasis of MDA-MB-231 cells overexpressing GAS7 or control were monitored by bioluminescence imaging (BLI) at day 55 after the implantation. Right: quantification results of primary tumors by BLI measurements were shown by dot plot. The Student’s *t*-test was used to compare these two groups (***p < 0.001). **c** The mRNAs from individual mouse lungs as shown in **a** and **b** were extracted, and analyzed for the levels of human-specific GAPDH and β-actin by RT-qPCR to quantify lung metastasis of MDA-MB-231 cells with GAS7b or control vector expressions. Student’s *t*-test was used to compare these two groups (***p < 0.001). **d** The MDA-MB-231-IV2 cells stably expressing GAS7b or control vector were orthotopically implanted into mammary fat pads of SCID mice (*n* = 5, each group). The axillary lymph-nodes from each mouse were removed at day 56 as shown. **e** The sizes of the primary tumors injected with MDA-MB-231-IV2 cells as in **d** were measured twice a week until day 56 post implantation, and the growth curves are shown (*n* = 5, each group). Linear regression model was used for statistical analysis, and showed no statistically significant differences (*p* = 0.395)

To evaluate whether GAS7b is functioning similarly in vivo, mouse model was employed. MDA-MB-231 cells with luciferase protein expressing were orthotopically implanted into mammary fat pads of SCID mice. The growth curve of tumors at the primary sites showed that the cancer cells stably expressing GAS7b grew significantly slower than the control group (Fig. 5a). We also monitored tumor growth via bioluminescence imaging (BLI) on day 10, 37, and day 55 after implantation (Supplementary Figure S10A, and Fig. 5b). BLI analysis indicated that both the size and weight of primary tumors were significantly reduced in the GAS7b overexpressing group than that in the control group (Fig. 5b and Supplementary Figure S10B). Western blotting analysis of the primary tumors confirmed the expected higher expression level of GAS7b and p27 in the GAS7b overexpressing group (Supplementary Figure S10C). We also examined the lung metastases by quantification of human-specific GAPDH mRNA levels in the mouse lung tissues. The RT-qPCR results showed that lung metastases were dramatically reduced in GAS7b overexpressing group as compared to the control group (Fig. 5c). Moreover, the ex vivo BLI analysis also revealed that mice injected with cancer cells overexpressing GAS7b had lower lymph-node metastasis as compared to the control mice (2/6 versus 5/5 of mice, respectively) (Supplementary Figure S10D). We also assessed the role of GAS7b in the highly invasive MDA-MB-231-IV2 subline by orthotopic implantation in mouse model, and the results demonstrated that MDA-MB-231-IV2 cells overexpressing GAS7b had reduced lymph-node metastasis (Fig. 5d), but there was no inhibition of tumor growth at the primary site (Fig. 5e and Supplementary Figure S11). These results indicated that GAS7b functions as a suppressor in growth and metastasis of breast cancer cells.

### *GAS7b* gene expression is transcriptionally regulated by p53

**Fig. 6:**
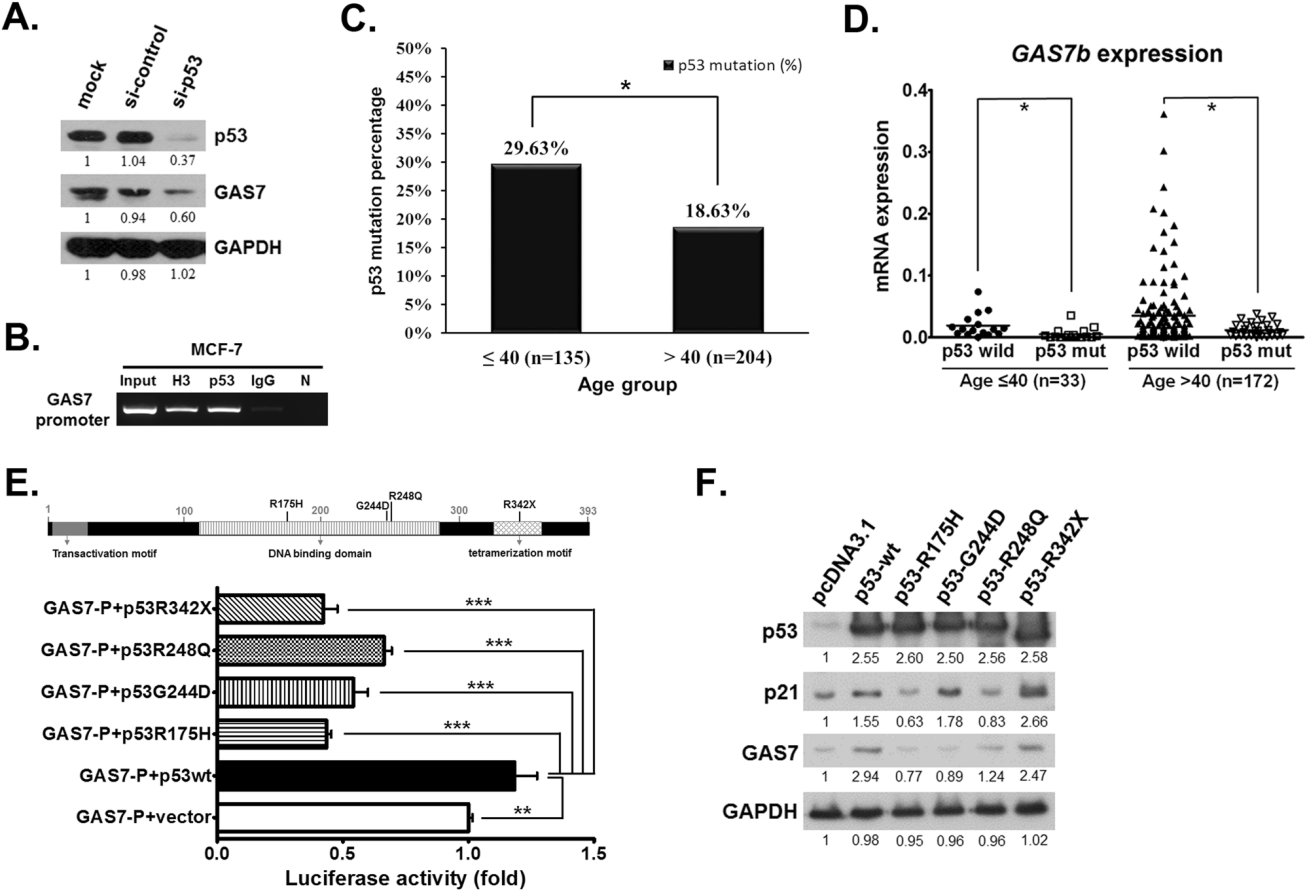
The *GAS7* gene expression was transcriptionally regulated by p53. **a** MCF-7 cells were transfected with control or GAS7 siRNA for 48 h, and the protein lysates were analyzed by western blotting using anti-p53, anti-GAS7, and anti-GAPDH antibodies. **b** ChIP assay was performed to detect p53 putative binding sites on the GAS7 promoter in MCF-7 cells. The 2% of total lysate DNA (input), DNA pulled-down by using histone H3 antibody (H3), p53 antibody (p53), IgG, or no antibody (N) were amplified using primers for putative p53 binding region in the GAS7 promoter. The PCR products were analyzed in 1.5% agarose gel as shown. **c** Genomic DNA from 339 breast cancer patients (including age ≤ 40 years old, *n* = 135; age > 40 years old, *n* = 204) were analyzed for the mutation of *p53* gene by Sequenom MassArray platform or whole exon sequencing technology. The *χ*^2^ test statistics analysis was used (*p < 0.05). **d** *GAS7b* mRNA expression was analyzed by RT-qPCR for the breast cancer patients with wild-type (*n* = 40) or mutant *p53* gene (*n* = 39). The Student’s *t*-test was used to compare these two groups (*p < 0.05). **e** Upper: schematic diagram illustrating the protein domains of p53 gene, and four mutation hotspots as indicated were found in our analysis. Bottom: dual-luciferase reporter assay showing MCF-7 cells co-transfected with PGL4-GAS7 promoter and PGL4-Renilla luciferase plasmid as internal control, and subsequently transfected with empty vector, wild-type p53, R175H, G244D, R248Q, or R342stop of p53 mutant expressing plasmid. Histograms represent normalized mean ± SD (*n* = 3). **f** Western blotting was performed to detect the expression of GAS7 and p21 under the regulations by wild-type or mutant p53 in MCF-7 cells. GAPDH protein was served as the loading control

To identify the potential upstream regulator for *GAS7* gene expression in breast cancer cells, we applied PROMO website to predict possible transcription factors of *GAS7* [16, 17]. A total of 40 putative binding sites of p53 were identified in the *GAS7* promoter and exon 1 regions (−800 bp to +350 bp from the *GAS7* transcription start site) (Supplementary Figure S12). To test the role of p53 in *GAS7* gene regulation, we knocked down p53 in MCF-7 cells, and observed a decrease in GAS7 expression (Fig. 6a). We further performed the experiment with MCF-7 cells treated pharmacological inhibitor of p53, Pifithrin-α (PFTα), which has been reported to inhibit p53 function and transactivation of its responsive genes [18]. The result showed that GAS7 expression was greatly decreased upon inhibition of p53 (Supplementary Figure S13). These results suggest that p53 is an important regulator for GAS7 expression. To verify this, the chromatin immunoprecipitation (ChIP) assay was performed with PCR primers flanking the region of the binding sites as shown in Supplementary Figure S12. We found that p53 directly bound to the *GAS7* promoter region (Fig. 6b). These data indicated that p53 directly bound to the *GAS7* promoter and regulated *GAS7* expression.

Next, we wanted to evaluate the correlations between mutation status of *p53* and expression of *GAS7b* in clinical samples. We performed DNA mass spectrometry for high throughput analysis for *p53* mutation sites, and whole *p53* exon sequencing, to analyze tumor DNA samples from a total of 339 Taiwanese breast cancer patients. The result showed that 81 patients were found to have mutations on 60 different sites in *p53* gene (Supplementary Table S3). Based on the age of initial diagnosis, we found that *p53* mutation rate was significantly higher in the group of patients aged below 40 years than that in the older age group (29.63% versus 18.63%) (Fig. 6c). Subsequently, we compared *GAS7b* expression levels between the two age groups of patients with wild-type and mutated *p53*. In both younger and older age groups, we found that patients with *p53* mutations had significantly lower *GAS7b* levels than patients with wild-type *p53* (Fig. 6d), suggesting that *p53* mutation was associated with downregulation of *GAS7b* expression in breast cancer, and this is in agreement with the transcriptional regulation of *GAS7* by p53 described above.

To assess whether *p53* mutation impacts *GAS7* expression, we constructed a reporter plasmid with *GAS7* promoter (Supplementary Figure S12), and four expression plasmids with *p53* mutations at the sites of R175H, G244D, R248Q in DNA binding domain, and R342stop in the tetramerization motif, where the higher mutation rate was found in our study (Supplementary Table S3). Luciferase activity assays demonstrated that *GAS7* promoter activity was decreased from all mutated *p53* gene comparing to the wild-type *p53* (Fig. 6e). Furthermore, we examined the effect of mutated p53 gene on regulating GAS7 and p21 expression in MCF-7 cells, and we found that GAS7 expression was lower as expected, and was correlated with mutant p53 genes, especially with R175H, G244D, and R248Q mutations (Fig. 6f). Overall, our observations indicate that p53 binds to *GAS7* promoter and promotes its transcription. Certain *p53* mutations impair this activity of p53, suggesting that the higher *p53* gene mutation rate in the younger breast cancer patients might in part account for the lower *GAS7* expression.

### Higher GAS7 expression is correlated with decreased tumor metastasis and better survival

**Fig. 7:**
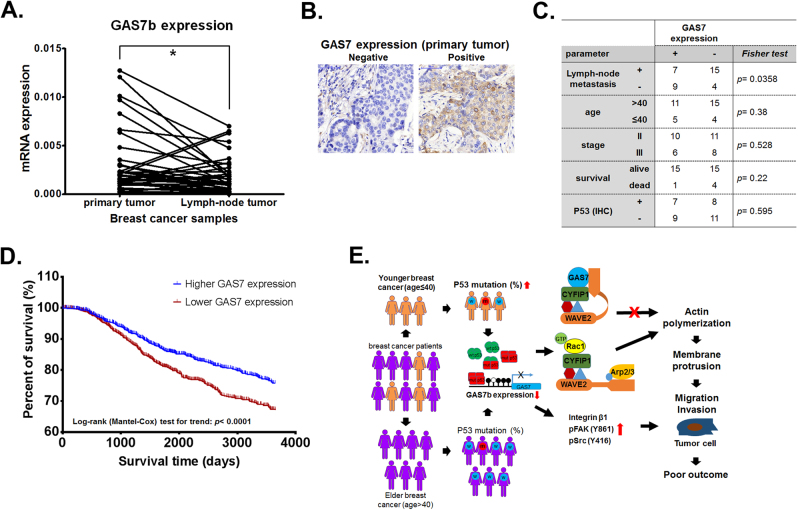
The GAS7 expression is correlated with tumor metastasis and patients survival. **a** RT-qPCR analysis for *GAS7b* mRNA expression levels in paired primary and lymph-node metastatic tumors from 38 breast cancer patients. The paired *t*-test was used for statistical analysis (*p < 0.05). **b** Immunohistochemistry (IHC) analysis was performed to detect the GAS7 expression in commercial breast tumor tissue array. Representative graphs (400×) of IHC staining of negative and positive staining are shown. **c** The GAS7 expression levels derived from IHC in relation to clinical parameters of the cases from commercial breast tumor tissue array. The Fisher’s exact test was used for the statistical analysis. **d** Kaplan–Meier survival plot analysis for the *GAS7* mRNA expression level in relation to 10-year overall survival in Curtis breast cancer data set. Patients were grouped into higher (*n* = 986) and lower (*n* = 986) *GAS7* expression for the analysis. Log-rank test (Cox proportional hazard model) was used for statistical analysis (hazard ratio = 0.7381, *p* < 0.001). **e** The model of the role of GAS7 and its involved pathway in regulation of cancer metastasis in early onset breast cancer

To determine whether there was an association between *GAS7* expression levels and clinical parameters in breast cancer patients, the mRNA samples from 38 pairs of primary and lymph node metastatic tumors, as well as commercial breast cancer tissue arrays, were analyzed by RT-qPCR and immunohistochemistry (IHC) assays, respectively. We found significantly lower *GAS7b* mRNA expression level in the lymph-node metastatic tumors compared to their paired primary tumor samples (Fig. 7a), For IHC analysis, we used commercial tissue array to analyze GAS7 protein expression in clinical breast tumor samples (Fig. 7b). The result indicated that lower GAS7 expressions were significantly correlated with positive lymph-node metastasis (Fig. 7c). These results are consistent with the findings in our mouse model experiments.

To further evaluate the clinical significance of *GAS7* in breast cancer patients, several public domain of breast cancer data sets derived from gene expression microarray analyses were investigated. We examined the *GAS7* expression in the breast cancer patients from Curtis breast data set [19], from which 1972 patients with 10-year survival record were selected. These patients were further divided into higher and lower groups according to the median *GAS7* expression level. The Kaplan–Meier survival plot indicated that the group of patients with higher *GAS7* expression had better 10-year survival as compared to the group with lower *GAS7* expression which had the worse clinical outcome (hazard ratio = 0.7381, *p* < 0.001) (Fig. 7d). Furthermore, the analysis from Bild breast data set (GEO accession: GSE3143) [20] showed that the breast cancer patients having higher *GAS7* expression displayed better 5 years survival after initial diagnosis than the lower *GAS7* expression patients (Supplementary Figure S14A). Kaplan–Meier analysis also revealed significant difference in overall survival between *GAS7*-high and *GAS7*-low expression groups (Supplementary Figure S14B). Moreover, the Schmidt breast cancer data set (GEO accession: GSE11121) [21] showed that the trend of lower *GAS7* expression was associated with overall metastatic events (Supplementary Figure S14C), as well as metastasis within 5 years (Supplementary Figure S14D). The analysis from data sets indicates that lower *GAS7* expression level is correlated with breast cancer metastasis. The phenomenon is consistent with our observations in cell and animal models, as well as with our analysis of clinical specimens (Figs. 2, 5 and 7). Overall, our study suggests that *GAS7* could be a useful biomarker for early onset, as well as for metastasis and survival of breast cancer.

## Discussion

Breast cancers in younger women have been correlated with more aggressive clinical behavior and inferior survival when compared with the older counterparts [22–25]. In previous studies, it has been reported the breast cancer patients diagnosed at <40 years old are more likely to have worse clinic-pathological features and more aggressive subtypes. For younger age group of breast cancer patients, there is a higher frequency of grade 3 tumors, higher lymphatic invasion, lower estrogen receptor (ER) and progesterone receptor (PR) positivity, higher human epidermal growth factor receptor 2 (HER-2) expression, and larger tumors [1, 26, 27]. These pathological features are most likely the reasons for poor survival of the younger age group of breast cancer patients.

In this study, we found that lower GAS7 expression significantly correlated with not only the early onset breast cancer, but also with tumor metastasis and patient survival (Fig. 7 and Supplementary Figure S14), suggesting GAS7 may play a role in tumorigenicity and aggressiveness in early onset breast cancer.

The *gas7* gene was first identified as one of the genes which were activated during growth arrest of NIH3T3 fibroblasts [28, 29]. Our results are consistent with the earlier reports in that we found GAS7b could regulate MDA-MB-231 and MCF-7 breast cancer cells proliferation in the in vitro and animal models (Supplementary Figures S3B, S10 and Fig. 5). However, our data showed GAS7b did not inhibit MDA-MB-231-IV2 proliferation in primary tumor growth in the orthotopic mouse model (Supplementary Figure S11), similar to that observed in PC12 cells [30]. We suspect MDA-MB-231-IV2 cells that have been derived from in vivo selection of mouse lung metastases of parental MDA-MB-231 cells [13] and have a more aggressive phenotype, are presumably due to activation of certain oncogenic pathways that are capable of overcoming the GAS7 inhibition of cell proliferation, but not invasive ability.

The expression of *GAS7c* also showed a downward trend in breast tumors (Fig. 1a, c), although not as significant as *GAS7b*. Our preliminary study indicated that the functions of GAS7c were similar to GAS7b in the inhibition of proliferation and metastasis of breast cancer cells (Supplementary Figure S15). These results are consistent with a recent study in lung cancer showing that the GAS7c acts as a metastasis suppressor [31]. Thus, *GAS7b* and *GAS7c* may function as suppressor in different types of cancer.

The protein sequence indicates GAS7 belongs to a subfamily of F-BAR domain protein. The GAS7b protein has a WW domain in its N-terminal region, and F-BAR domain in the center region, but lack of the SH3 domain compared with GAS7c. The F-BAR domain proteins have been shown to be important coordinators in membrane curvature regulation, including mechanism of cytokinesis, endocytosis, phagocytosis, as well as the formation of filopodium, lamellipodium, adhesion, and podosome [32]. For example, CIP4 protein inhibits neurite formation by producing lamellipodium, and this effect depends on the F-BAR and SH3 domains [33]. Breast cancer cells with CIP4 knockdown displayed increased numbers of mature invadopodia, and the cells were shown to be more invasive [34]. Here, we found that overexpression of GAS7b disrupted the spreading of cells, and reduced the number of focal adhesion complexes (Fig. 2d, e). We also showed that GAS7b could downregulate Rac1-GTP and inhibited cell migration and invasion (Fig. 4b–d). These functions of GAS7b appear to be similar to CIP4 protein, which acts as effectors regulating Rac1-mediated cell spreading and migration.

A study showed that GAS7b interacted with N-WASP to regulate the neurite outgrowth in hippocampal neurons. Although GAS7b lacks the SH3 domain, the WW domain instead may mediate the protein–protein interaction [35]. Here, we propose a regulatory mechanism of breast cancer cell migration in that GAS7b interacts with CYFIP1 protein and prevents recruitment of Rac1-GTP to block actin polymerization, leading to the inhibition of motility of breast cancer cells.

Breast cancer studies have demonstrated that 18.5–22.8% mutation rate on *p53* gene were found in Taiwanese breast cancers [36–38]. We observed overall of 23.9% total mutation rate on *p53* gene in our study cohort, which is similar to the previous studies. In particular, our study is the first to identify that there is a significantly higher somatic *p53* mutation rate in the early onset than late onset breast cancer patients (29.63% and 18.63%, respectively) (Fig. 6c). Previous study has shown that germline *TP53* gene mutations could predispose patients to early onset breast cancer [39]. Therefore, we analyzed 29 paired normal tissue DNAs from the breast cancer patients whose tumor tissues have *p53* gene mutations. The result indicated that there were no mutations in the *p53* gene in the normal tissues from breast cancer patients (data not shown), suggesting at least in our cases, breast cancer tissue mutations are somatic rather than germline mutations. However, the detailed mechanisms of higher p53 mutations in the early onset breast cancer is still unclear. We speculate that it may be related to the ways of early onset carcinogenesis including environmental pollutants or lifestyle, such as alcohol and tobacco exposure. Further study is needed to explore the underlying mechanism. In addition, the heritable vulnerability trait may be another reason of higher p53 mutation in the early onset breast cancer.

Previous studies showed that chromosome deletion and promoter methylation could be the reasons for lower GAS7 expression in lung cancer [31, 40]. Therefore, we performed the methylation-specific PCR (MSP) to detect the promoter methylation of *GAS7*. The preliminary results indicated that several breast cancer cell lines as well as many clinical tumor samples display hypermethylation in the *GAS7* promoter region (Supplementary Figure S16), suggesting that promoter methylation could be another mechanism for regulating *GAS7* gene expression. However, we found that there was no correlation between *GAS7* promoter methylation and patients’ age of breast cancer initial diagnosis (data not shown).

In conclusion, our study shows higher *p53* mutation rate in early onset breast cancer patients, who have significantly reduced *GAS7* expression. The GAS7b functions as a metastasis suppressor at least in part via inhibition of the actin polymerization through CYFIP1/GAS7 protein complex mediated binding of GTP-Rac1 and inhibition of integrin-mediated FAK–Src–Rac1 signaling. Our analysis of clinical samples and public data reveal an association of lower *GAS7* expression with breast cancer metastasis and poor survival (Fig. 7e). Therefore, *GAS7b* could serve as a biomarker for tumor metastasis and prognosis in early onset breast cancer patients.

## Materials and methods

### Clinical sample preparation and DNA/RNA extraction

Clinical tissues from breast cancer patients were collected from National Taiwan University Hospital, Chi-Mei Medical Center, Chia-Yi Christian Hospital, and Kaohsiung Medical University Chung-Ho Memorial Hospital. The DNA/RNA extraction procedures are described in Supplementary Materials and Methods.

### Exon array analysis

The 25 pairs of breast normal/tumor tissue specimens were analyzed by Affymetrix Human Exon 1.0 ST Array (Affymetrix, Santa Clara, CA). The detailed analysis procedures are described in Supplementary Materials and Methods.

### Quantitative real-time PCR

Primer sequences and the conditions used for quantitative real-time-PCR are described in Supplementary Materials and Methods and Table S1.

### Cell lines and culture conditions

The culture conditions of MCF-10A, MDA-MB-231, MDA-MB-231-IV2, Hs578T, and MCF-7 cell lines are described in Supplementary Materials and Methods.

### Transwell cell migration and invasion assay

Cell migration and invasion assays were carried out as described previously [41]. The detailed assay procedures are described in Supplementary Materials and Methods.

### Wound healing migration assay

Wound healing migration assays were performed according to the methods indicated in our previous study [41]. The detailed procedures are described in Supplementary Materials and Methods.

### Cell adhesion assay

Detailed procedures are described in Supplementary Materials and Methods.

### Focal adhesion, F-actin immunofluorescent staining, and confocal microscopy assay

The assay procedures were similar to those described previously [41]. The detailed procedures and antibodies used for staining are described in Supplementary Materials and Methods.

### Western blot analysis

Detailed procedure and antibodies for blotting are described in Supplementary Materials and Methods.

### Co-immunoprecipitation (co-IP) assay

Detailed procedure and antibodies for IP and blotting are described in Supplementary Materials and Methods.

### Pull-down assay of activated Rac1

MDA-MB-231 cells were transfected with GAS7b expressing or control empty vector, and subsequently transfected with GAS7 or control siRNA (Table S1). After 48 h of incubation, cells were analyzed for activated Rac1 as described previously [42].

### Orthotopic mouse model assay

We established MDA-MB-231 and MDA-MB-231-IV2 cells that were stably transfected with GAS7b, GAS7c expressing, or control empty vector. Detailed procedures are described in Supplementary Materials and Methods.

### Chromatin immunoprecipitation (ChIP) and target region ChIP-PCR

ChIP assay was carried out using the Chromatin Immunoprecipitation Kit (17-408, Millipore) following the manufacturer’s manual. The detailed procedures, antibodies, and primer sequences are described in Supplementary Materials and Methods and Table S1.

### The p53 gene mutations assay

Genomic DNAs from the 257 breast cancer samples that we had procured were analyzed for the mutations of *p53* gene by MassArray platform (Sequenom, San Diego, CA), and the whole exon sequencing data from another 82 young breast cancer patients were analyzed for the mutations of *p53* gene. The detailed information is described in Supplementary Materials and Methods.

### Immunohistochemistry (IHC) assay

Commercial breast tissue arrays were purchased from SUPER BIO CHIPS (Seoul, South Korea), detailed procedures and GAS7 antibody for staining are described in Supplementary Materials and Methods.

## Electronic supplementary material

supplementary information
