# Supplementary material for: Wild-type p53 upregulates an early onset breast cancer-associated gene GAS7 to suppress metastasis via GAS7–CYFIP1-mediated signaling pathway
Source: Oncogene. 2018 Apr 30;37(30):4137–50. doi: 10.1038/s41388-018-0253-9 (PMC6062498; doi:10.1038/s41388-018-0253-9)
Supplement: Supplementary file 1 — supplementary information [file 41388_2018_253_MOESM1_ESM.pdf]

Wild type p53 upregulates an early onset breast cancer associated gene GAS7 to suppress metastasis via GAS7-CYFIP1 mediated signaling pathway

Jer-Wei Chang, Wen-Hong Kuo, Chiao-Mei Lin, Wen-Ling Chen, Shih-Hsuan Chan, Meng-Fan Chiu, I-Shou Chang, Shih-Sheng Jiang, Fang-Yu Tsai, Chung-Hsing Chen, Pei-Hsin Huang, King-Jen Chang, Kai-Ti Lin, Sheng-Chieh Lin, Ming-Yang Wang, Yih-Huei Uen, Chi-Wen Tu, Ming-Feng Hou, Shih-Feng Tsai, Chen-Yang Shen, Shiao-Lin Tung, Lu-Hai Wang*

Supplemental Materials and Methods

Clinical sample preparation and DNA/RNA extraction

Clinical tissues from breast cancer patient were collected from National Taiwan University Hospital, Chi-Mei Medical Center, Chia-Yi Christian Hospital, and Kaohsiung Medical University Chung-Ho Memorial Hospital. The genomic DNA was prepared by proteinase K digestion, followed by phenol-chloroform extraction. Total RNA was prepared from tumors and normal breast tissues by using TRIzol reagent (Invitrogen, Carlsbad, CA).

Exon-array analysis

The total RNA of 25 sets of normal/ tumor pair of breast tissue specimens was extracted, and analyzed by Affymetrix Human Exon 1.0 ST Array (Affymetrix, Santa Clara, CA). The raw data was normalized and analyzed by GeneSpring software (Agilent Technologies, Santa Clara, CA), followed by Gene Ontology (GO) annotation analysis, and classified into GO molecular function domains. The exon-array data of 25 sets of normal/ tumor pair were uploaded to Gene Expression Omnibus (GEO) database (https://www.ncbi.nlm.nih.gov/geo/), and the accession codes is GSE109169.

Public domain datasets analysis

The public cancer microarray datasets from Oncomine database (http://www.oncomine.org) [1] was used to validate the candidate genes from our exon-array analysis and GAS7 gene expression was found to be correlated with clinical parameters. Several datasets were used in this study, including Curtis breast dataset (EGA: EGAS00000000083) [2], Bild breast dataset (GEO: GSE3143) [3], Schmidt breast dataset (GEO: GSE11121) [4], Pawitan breast dataset (GEO: GSE1456) [5], and TCGA breast dataset (data version: 2011/09/02 release) (http://cancergenome.nih.gov/).

Quantitative real-time PCR (qRT-PCR)

The cDNA samples were synthesized by using SuperScript III Reverse Transcriptase (Invitrogen). The qRT-PCR was performed by using KAPA SYBR FAST Universal master mix (KAPA Biosystems, Wilmington, MA) with CFX96 real-time PCR detection system (Bio-Rad, Hercules, CA) for gene expression detection. The results were normalized with β-actin or GAPDH expressions. Detailed primer sequences used for qRT-PCR are provided in Table S1.

Cell lines and culture conditions

Non-tumorigenic mammary epithelial line MCF-10A (American Type Culture Collection, ATCC) was cultured in DMEM/F-12 medium (GIBCO, Grand Island, NY) supplemented with 5% of horse serum (GIBCO), 20 ng/ml of EGF (ProSpec-Tany TechnoGene Ltd., Rehovot, Israel), 10 μg/ml of insulin (Sigma-Aldrich, St. Louis, MO), 0.5 μg/ml of hydrocortisone (Calbiochem, Darmstadt, Germany), 100 ng/ml of cholera toxin (Sigma-Aldrich), and 1% of penicillin-streptomycin (GIBCO). Human breast cancer cell lines, MDA-MB-231, Hs578T and MCF-7 (ATCC) were cultured in DMEM medium (GIBCO) containing 10% of fetal bovine serum (Biological Industries, Israel) and 1% of penicillin-streptomycin (GIBCO). MDA-MB-231-IV2 cell line was *in vivo* selected from

MDA-MB-231 injected SCID mice cells, which has shown to have higher metastatic capability compared with the parental MDA-MB-231 cells in our previous study [6]. The culture condition for MDA-MB-231-IV2 was the same as MDA-MB-231 cells. The MDA-MB-231 cells stably transfected with pcDNA3.1-GAS7b or empty vector were established by G418 (Sigma-Aldrich) selection, and subsequently transfected with pLAS5w-Luc-2A-eGFP plasmid via lentivirus infection system and selected with puromycin (Sigma-Aldrich). These cells were able to stably express both green fluorescent protein and luciferase protein. All of the cell lines were incubated at 37°C in a humidified, 5% CO_2 incubator.

Cell line transient transfection

TransIT-2020 (Mirus, Madison, WI) and Lipofectamine RNAiMAX transfection reagent (Thermo Fisher Scientific, Waltham, MA) were used for transient transfection of cells with expression vectors and siRNAs, respectively. All the experimental protocols followed the manufacturer’s manual.

Cell proliferation assay

The $5x10^3$ of cells were seeded into each well of 12 well culture plates, and

incubated at 37°C in humidified, 5% CO_2 incubator. After 24 hours of incubation, 100μl of CellTiter 96® AQueous One Solution Reagent (Promega, Madison, Wisconsin) was added into the well containing the cells and 500μl of culture medium, the plate was then further incubated for 2 hours in the incubator. The 100μl reaction medium from each well was transferred into a 96 well plate after the incubation, and the absorbance at 490 nm was measured with an ELISA reader. This assay was repeated daily for 4 days after seeding of the cells.

Transwell cell migration and invasion assay

The cell migration and invasion assays were conducted with 24-well format of Transwell inserts (Falcon 353097, and Corning BioCoat 354480) by seeding of $5x10^4$ cells in 0.1% BSA-containing D-MEM per well, and with 10% FBS-containing DMEM in the lower chamber of the plate, followed by incubation for 8 hours for migration assay and 22 hours for invasion assay, respectively. After the incubations, cells on the filter membrane were fixed and stained by PBS solution containing 10% methanol, 1% formaldehyde, and 0.1% crystal violet for 10 mins. Pictures of five different regions on the filter membrane were taken under microscope, and the number of cells was quantified by using Image J software (NIH, USA).

Wound healing migration assay

The wound healing migration assays were performed according to the methods as described in our previous study [7]. Briefly, MDA-MB-231 cells were transfected with empty or GAS7b plasmid, and adjusted to a concentration of $5x10^5$ cells/ml. The cell suspension in 70 µl was transferred into each well of culture inserts (Ibidi, Martinsried, Germany) which were placed in a 6 well cell culture plate, and incubated at 37 °C with 5% CO_2 incubator for 24 hours. The culture inserts were removed after the incubation, and 2 ml of culture medium was added back into each well of the 6-well culture dish. The cells were further incubated for 24 hours and photographed once every 30 minutes by a live imaging Leica AF 6000 LX microscope (Leica, Wetzlar, Germany) during the incubation. The migrated cells were quantified using MetaMorph image analysis software (Molecular Devices, Sunnyvale, CA).

Live cell imaging assay

The microscopic live cell imaging assay was performed to monitor the migratory distance of cells. The MDA-MB-231-IV2 cells were transfected with empty or GAS7b plasmid, and 5×10^4 cells were seeded into a 6 well plate. After cells

were attached, they were photographed once every 5 minutes by a live imaging Leica AF 6000 LX microscope (Leica) during the following 24 hours of incubation. The magnification of the microscope is 100x. The overall migratory distance of cells was quantified by MetaMorph image analysis software (Molecular Devices).

Cell adhesion assay

A total of 5×10^5 MDA-MB-231 or MDA-MB-231-IV2 cells transfected with empty or GAS7b plasmid were seeded onto a fibronectin (Millipore, Billerica, MA), type I collagen (Millipore, Billerica, MA) or Laminin (Corning, New York) pre-coated 6 wells plate, followed by incubation for 1 hour in 37°C incubator. Cells were then rinsed for three times with PBS, and fixed with 3.7% formaldehyde for 10 minutes at room temperature. The cells were stained with crystal violet solution for 10 minutes after the fixation, they were then washed five times with ddH_2O. The plate was air dried, and 1 ml of 33% acetic acid was added into each well to dissolve the crystal violet, and dye solution was measured at OD.550 nm by a spectrophotometer.

Focal adhesion (FA), F-actin immunofluorescent staining and confocal

microscopy assay

The procedures were similar as described previously [7]. In briefly, MDA-MB-231 and MDA-MB-231-IV2 cells were transfected with pcDNA3.1-GAS7b, pCMV-HA-GAS7b, or empty vector. Forty eight hours after transfection, the cells were trypsinized and seeded onto fibronection (10μ/ml; Millipore)-coated coverslips and incubated for 1.5, 4, 8, 12, and 24 hours, respectively, before fixation and immunofluorescence staining were performed. The focal adhesions of cells were detected with anti-vinculin (Sigma-Aldrich) or anti-paxillin (05-417; Millipore) antibodies, GAS7b-HA was detected with anti-HA (#3724; Cell Signaling Technology, Danvers, MA) antibody, Rhodamine Phalloidin (R415; Invitrogen) and DAPI were used for actin and nuclear staining respectively. Images were photographed using TCS SP5Ⅱ confocal microscopy (Leica, Wetzlar, Germany) with 63x objective lenses.

Western blot analysis

The cells were lysed with RIPA lysis buffer (with 0.1% SDS) containing 1x proteinase and phosphatase inhibitors (Roche, Basel, Switzerland), and protein samples were resolved in 10% SDS-PAGE. The proteins were then transferred onto PVDF blotting membrane (Millipore) and probed with antibodies. The

antibodies against the following proteins were used GAS7 (PA5-30274; Thermo Fisher Scientific), β1-integrin (sc-6622; Santa Cruz Biotechnology, Dallas, TX), p-FAK (Tyr397; ab81298; Abcam), FAK (05-537; Millipore), p-Src (Tyr416; #6943; Cell Signaling Technology), Src (#2109; Cell Signaling Technology), Rac1 (05-389; Millipore), p53 (sc-126; Santa Cruz Biotechnology), p21 (sc-6246; Santa Cruz Biotechnology), p27 (sc-528; Santa Cruz Biotechnology), CYFIP1 (AB6046; Millipore), WAVE2 (#3659; Cell Signaling Technology), β-actin (sc-56459; Santa Cruz Biotechnology) and GAPDH (sc-32233; Santa Cruz Biotechnology). GAPDH and β-actin were used as the loading controls.

Co-immunoprecipitation (Co-IP) assay

MCF-7 cells was transfected with GAS7 or control siRNA (Table S1), subsequently transfected with the constitutively active Rac1 (ca-Rac1) plasmid, and incubated for 48 hours. The cells were lysed with IP lysis buffer (100mM Na_2HPO_4, 150mM NaCl, 1mM $MgCl_2$, pH=7.2), and 1 mg of cellular proteins from the lysate was incubated with 5 ug antibodies against GAS7 (sc-365385; Santa Cruz Biotechnology), CYFIP1 (AB6046; Millipore), or control IgG, respectively, at 4°C for overnight. Subsequently 20μl of protein A or protein G agarose beads (Millipore) was added into the reaction and incubated for 1 hour

at 4°C. The agarose beads were washed three times with IP lysis buffer after the incubation, and 20ul of Laemmli protein sample buffer was added to each sample and boiled before subjection to PAGE and Western Blot analysis.

Orthotopic mouse model assay

MDA-MB-231 and MDA-MB-231-IV2 cell lines that stably expressing GAS7b, GAS7c, or control pcDNA3.1(-) vector were established. MDA-MB-231 stable cell line was transfected with pLAS5w-Luc-2A-eGFP plasmid via lentivirus infection system for stable expression of green fluorescent GFP and luciferase protein. The $1x10^6$ of cells re-suspended in 100 μl PBS containing 30% of Matrigel (BD Biosciences, Franklin Lakes, New Jersey) were orthotopically implanted into the 4th pair of mammary fat pads of CB17/Icr-Prkdscid/Crl female mice (BioLASCO, Taipei, Taiwan). Mice were grouped randomly for implanted the cancer cells, and tumor size was measured twice a week. The tumor growth and lymph node metastasis of MDA-MB-231 cells were also monitored by *in vivo* bioluminescence imaging (BLI) system (Caliper IVIS system; PerkinElmer) on day 10, 37, and 55 after implantation. The *ex vivo* imaging of various organs after sacrifice of the mice and harvesting of the organs was also monitored. The mice were sacrificed on Day 55 (MDA-MB-231) or Day 56 (MDA-MB-231-IV2)

post implantation. The primary tumors were removed and weighed, and proteins from the tumors were extracted by protein lysis buffer. The total mRNA was extracted from mouse lungs by TRIzol reagent (Invitrogen). Lung metastasis index was calculated using qRT-PCR by detecting human-specific GAPDH and normalized with universal β-actin. All procedures were approved by Institutional Animal Care and Use Committee (IACUC) of National Health Research Institute.

P53 inhibitor drug treatment

MCF-7 cells was treated with DMSO or 30μM of Pifithrin-α (PFTα) (P4359, Sigma-Aldrich), and incubated in 37°C incubator for 72 hours. The cells were lysed and performed with Western Blotting analysis.

Chromatin immunoprecipitation (ChIP) and target region ChIP-PCR

ChIP assay was carried out using the Chromatin Immunoprecipitation Kit (17-408, Millipore), following the manufacturer's manual. Briefly, MCF-7 cells were grown in a 100 x 20-mm culture dish to approximately 80–90% confluent, cells were cross-linked with 1% formaldehyde and then lysed. The lysates were sonicated using Bioruptor system (Diagenode, Liège, Belgium) to shear DNAs

to lengths between 200 and 800 bp. Subsequent steps were performed with the assay kit. IP was performed using anti-acetyl histone H3 (06-599B; Millipore), anti-p53 (sc-126; Santa Cruz Biotechnology) antibodies and mouse IgG. The ChIP-PCR primer was designed for the GAS7 promoter region. The primer sequences was listed in supplementary Table S1. The 1% of total input DNA and IP DNA samples were subjected to PCR, and PCR products were analyzed with 1.5% agarose gel.

The p53 gene mutations assay

Genomic DNA from 257 breast cancer patients (including age ≤ 40 years old, n=53; age > 40 years old, n=204) were extracted and analyzed for the mutations of p53 gene by Sequenom MassArray platform (Sequenom, San Diego, CA), and he sequencing services were provided by Feng Chi Biotech (Taipei, Taiwan) and EffPha Corporate (Taipei, Taiwan). The specific sites on p53 gene that were analyzed by MassArray are shown in Table S2. In addition, the mutations on p53 gene from whole exon sequencing for 82 young breast cancer patients (age ≤ 40 years old) were also analyzed.

Site directed mutagenesis and promoter luciferase reporter assay

The wild type pcDNA3.1-p53 expression vector was obtained from Dr. Hsin-Ling Hsu (NHRI, Taiwan), and p53 gene ontaining mutation(s) in the mutation hotspots were generated by QuickChange site-directed mutagenesis kit (Stratagene, La Jolla, CA). The wild type p53 plasmid was used as the template to generate specific various mutations (R175H, G244D, R248Q and R342X) on p53 gene by using specific primers (see Table S1). The promoter sequences of GAS7 were isolated and cloned from MCF-10A normal breast cellular DNA. The primers used for PCR are shown in Table S1. The GAS7 promoter fragment was cloned into pGL4.20 luciferase reporter vector (Promega). The $2x10^4$ of MCF-7 cells were seeded in 12 well plate, and the cells were co-transfected with pGL4.20-GAS7 promoter vector, pGL4.73 (Renilla reniformis), and together with wild-type or mutant p53 expression vector by using TransIT-2020 transfection reagent (Mirus). After 48 hours, the expression of luciferase gene was normalized and determined by Dual-Luciferase reporter assay kit (Promega).

Immunohistochemistry (IHC) assay

Commercially available breast cancer tissue array were purchased from SUPER BIO CHIPS (Seoul, South Korea). IHC was performed by NHRI

Pathology Core Laboratory (Miaoli, Taiwan) and Bond biotech Incorporation (Taichung, Taiwan). Monoclonal anti-GAS7 Ab (1:200; OriGene, TA501756; Derwood, MD, USA) was used for detecting GAS7 protein expression on the tissue sections. The tumor surrounding stroma and epithelial cells are served as normal cell control in each tissue sample.

Methylation specific PCR (MSP) assay

The methylation status of the GAS7 promoter was determined by methylation-specific PCR (MSP) analysis. EZ DNA Methylation-Gold™ Kit (Zymo Research, Orange, CA) was used for bisulfite conversion of DNAs from cell lines or breast tumor samples. The U-primer and M-primer for the –108 to +27 bp (NM_201433) regions in GAS7 promoter were used for MSP assay, and the primer sequences were listed in Table S1. CpGenome Universal Methylated DNA (S7821; Millipore) and Unmethylated DNA set (S7822; Millipore) are served as positive controls for bisulfite conversion, and for U or M PCR reactions.

Statistics

The images of cells from trans-wells, immunofluorescence staining, and protein bands from western blots were analyzed and quantified with Image J software

(NIH). The GraphPad Prism software (GraphPad Software, CA) was used for generating graphs and two-tailed paired or unpaired *t*-test was performed for determining significance between the groups compared. Except where otherwise noted, data presented in figure are showed as mean ± SD. The R project (R Development Core Team) was used for Log-rank Mantel-Cox test, linear regression model, and χ^2 test statistics analysis. The Kaplan-Meier estimate was used for overall survival, and the log-rank test was used to compare the cumulative survival durations in different patient groups. In addition, Cox proportional hazard model was applied to assess the significant level of association between GAS7 expression and overall survival. All the statistics test methods are indicated in the figure legends. A *P* value of less than 0.05 is considered significant.

Study approval

The clinical samples from breast cancer patients' were collected for mRNA expression and DNA mutation analysis under the approval from Institutional Review Board from each hospital. The animal experiment protocols were reviewed and approved by the Institutional Animal Care and Use Committee of National Health Research Institutes, Taiwan.

Supplemental Figures

25 NT pairs of breast cancer patients

⬇

Exon array analysis

⬇

T test paired analysis (5895 genes)

⬇

1.5 fold down-regulated genes in tumor samples (919 genes)

⬇

Gene ontology analysis: biological process_cell cycle regulation (14 genes)

Gene symbol	Entrez gene ID	FC ([T] vs [N])	Gene description
ZAK	51776	-1.51	sterile alpha motif and leucine zipper containing kinase AZK
KAT2B	8850	-1.77	K(lysine) acetyltransferase 2B
TP63	8626	-2.91	tumor protein p63
PKD2	5311	-1.99	polycystic kidney disease 2
DST	667	-2.44	dystonin
SESN1	27244	-1.61	sestrin 1
PLAGL1	5325	-2.56	pleiomorphic adenoma gene-like 1
TCF7L2	6934	-1.95	transcription factor 7-like 2
SESN3	143686	-1.97	sestrin 3
HEPACAM	220296	-1.75	hepatocyte cell adhesion molecule
MAP2K6	5608	-1.57	mitogen-activated protein kinase kinase 6
GAS7	8522	-1.64	growth arrest-specific 7
PPP1R15A	23645	-1.50	protein phosphatase 1, regulatory (inhibitor) subunit 15A
PRNP	5621	-2.15	prion protein

⬇

Public domain datasets validation: analyzed these 14 genes in Curtis breast dataset (The 9 genes showed significant difference between cancer and normal samples)

⬇

The 3 genes expression pattern in Curtis breast dataset showed significant difference between ≤ 40 and 40-60 years old patients.

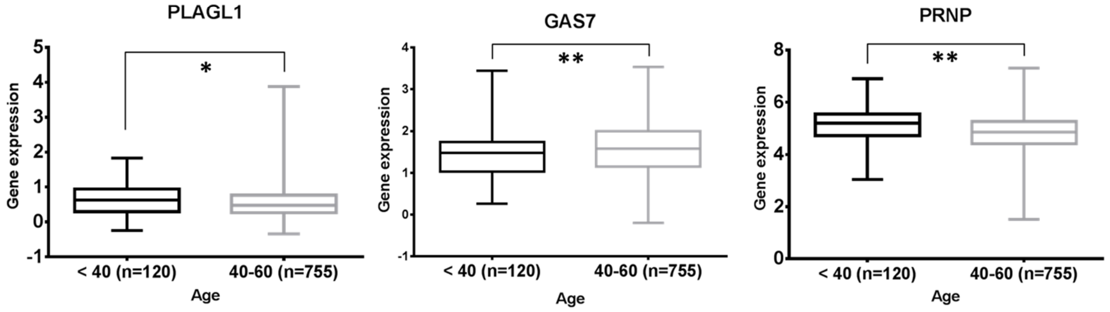

Figure S1. Flow diagram summarizing the gene expression data used for various analyses. The 25 sets of breast tumors and their normal pairs were analyzed by Affymetrix Human Exon 1.0 ST array, followed by standard gene array analytical methods. Based on a previous study, higher percentage of younger aged breast cancer patients have larger tumor size upon initial diagnosis in comparison with the older aged patients. Thus we were interested in searching for the involvement of the cell growth related genes in the early onset breast cancer. We identified 14 genes involved in cell cycle regulation to

be differentially expressed in tumor tissues. To assess whether those genes were involved in early-onset breast cancer, we subsequently analyzed the expression profiles of these genes in Curtis breast cancer dataset [2]. Nine genes were found to be differentially expressed between cancer and normal tissues (**Figure S2**), and three (*PLAGL1*, *GAS7*, and *PRNP*) of them showed statistically significant differences between breast cancer patients with age less than 40 years old, and those between 40 to 60 years old. The Oncomine® Platform (https://www.oncomine.org) was used to search for the breast cancer datasets in this study. The star symbol showed statistically significant differences (*t*-test, $^*p < 0.05$; $^{**}p < 0.01$).

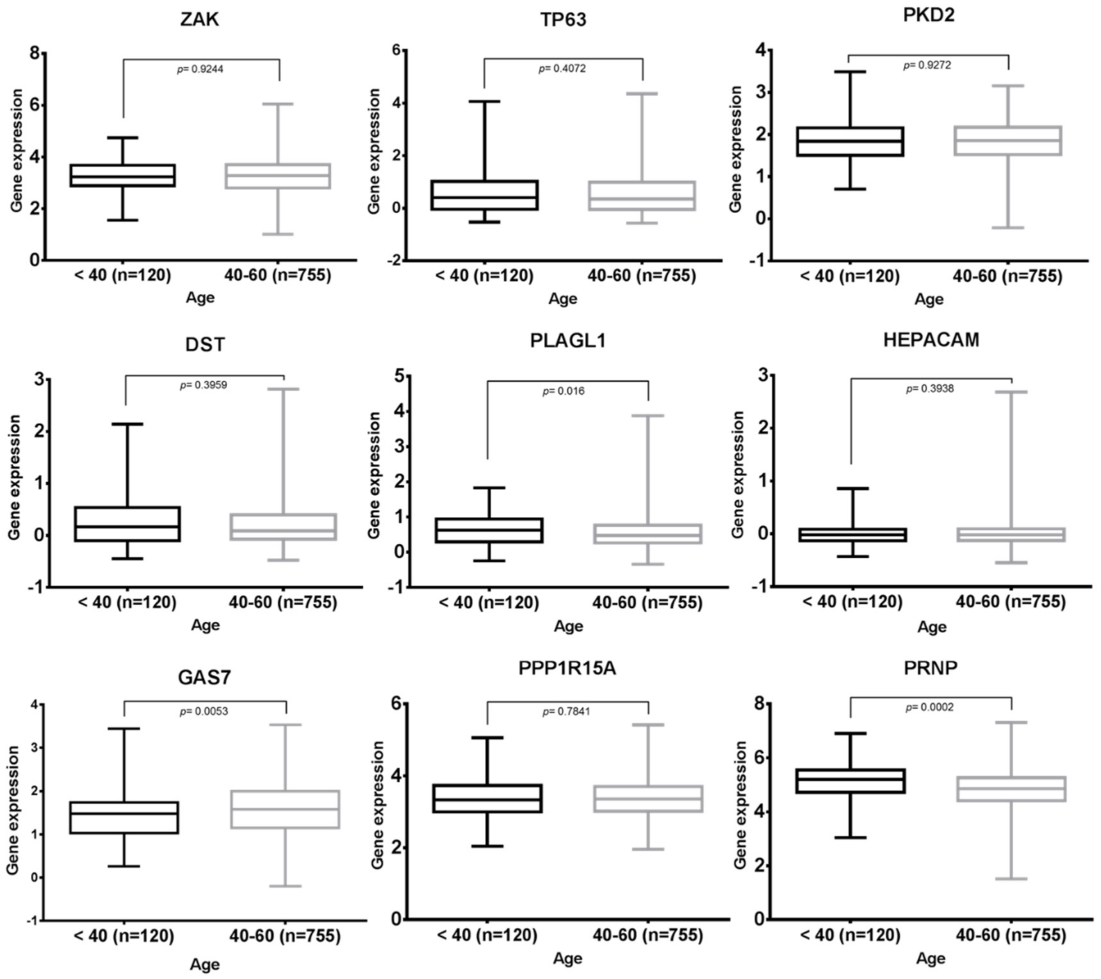

Figure S2. The expression profiles of 9 cell cycle related genes are stratified by different age group. The expression profiles of 9 genes (data from Curtis breast dataset) were compared for patients' aged ≤40 years old (n=120) and aged between 40 to 60 years old (n=755). Two-tailed *t*-test was used for statistical analysis. The $p < 0.05$ showed statistically significant differences.

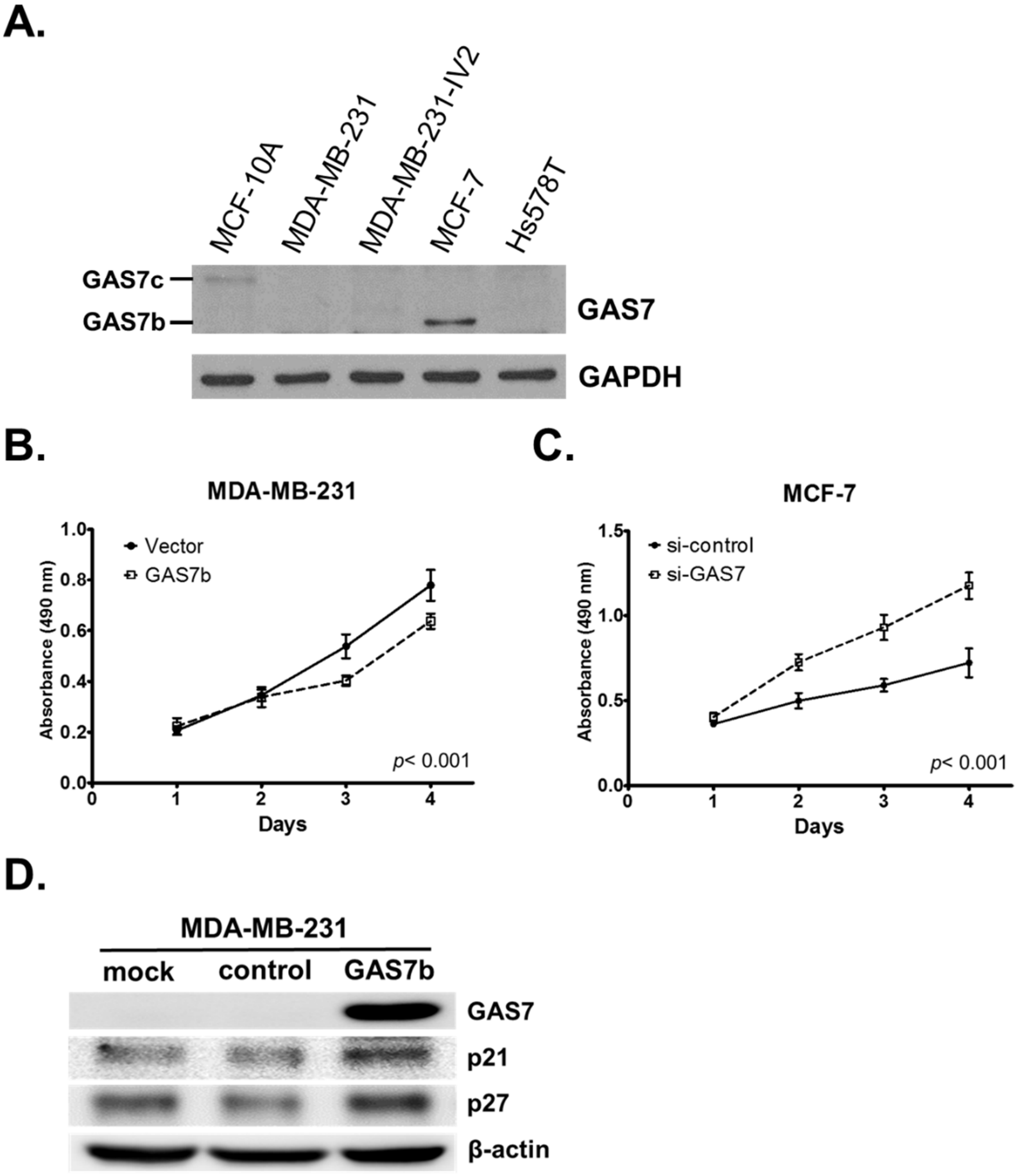

Figure S3. GAS7 regulates breast cancer cell proliferation. (A) Immunoblotting assay of GAS7 in breast normal (MCF-10A) and cancer (MDA-MB-231, MDA-MB-231-IV2, MCF-7, and Hs578T) cell lines. **(B)** MTS assays were performed to detect cell proliferation of overexpressing GAS7b in MDA-MB-231 cells, and **(C)** knockdown GAS7 in MCF-7 cells. The results were repeated in two additional independent experiments for the two cell lines. Statistical significance was calculated using linear regression model. **(D)** Immunoblotting analysis of GAS7, p21, and p27 from MDA-MB-231 cells transfected with control or GAS7b expression vector. The β-actin protein was served as the loading control.

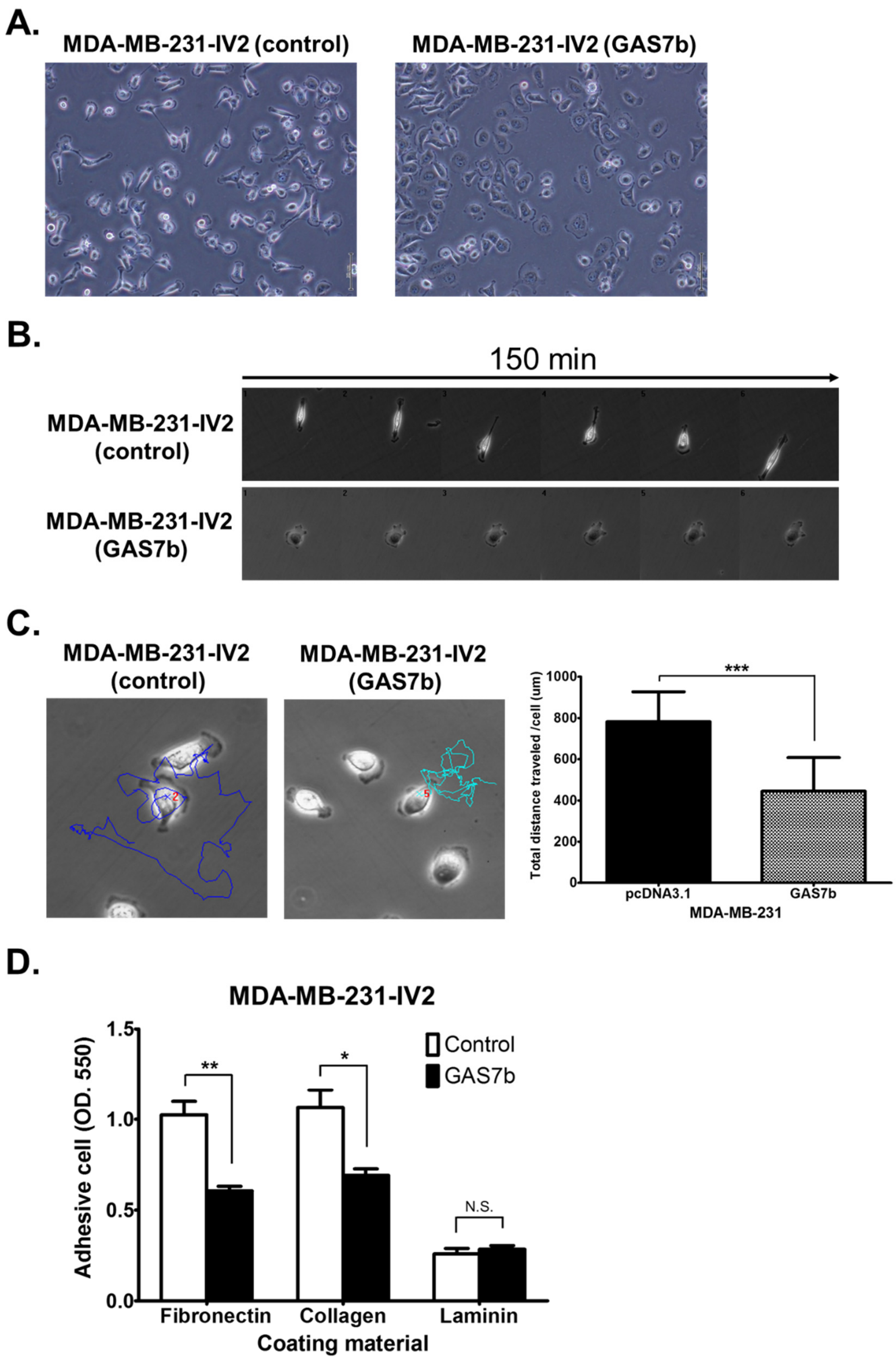

Figure S4. GAS7b affects the MDA-MB-231-IV2 cell shape and inhibits cell migration and adhesion. MDA-MB-231-IV2 cells were transfected with control or GAS7b expressing plasmid, and incubated for 72 hours **(A)** Microscopic photographs for the control and GAS7b expressing cells. **(B)** Time-lapse

photography applied to microscopy was used to monitor cell movement. The picture was taken once every 30 min during the assay. **(C)** Time-lapse analysis of cell migration was performed, and the picture was taken once every 5 min. Left, the lines represent the path of travel for 24 hours. Right, the traveling path of 10 cells from two groups were calculated for the mean of total distance traveled per cell. Histograms represent normalized mean±SD, and Student's *t*-test was used for statistical analysis (***$p < 0.001$). **(D)** The cell adhesion assay was performed with fibronectin, Type I collagen, or laminin coaed plates. The adhered cells were staining by crystal violet, and subsequently measured for retention of the dye by spectrophotometer. Data represent normalized mean±SD (n=3). Two-tailed *t*-test was used for statistical analysis (* $p < 0.05$; ** $p < 0.01$; n.s., non-significant).

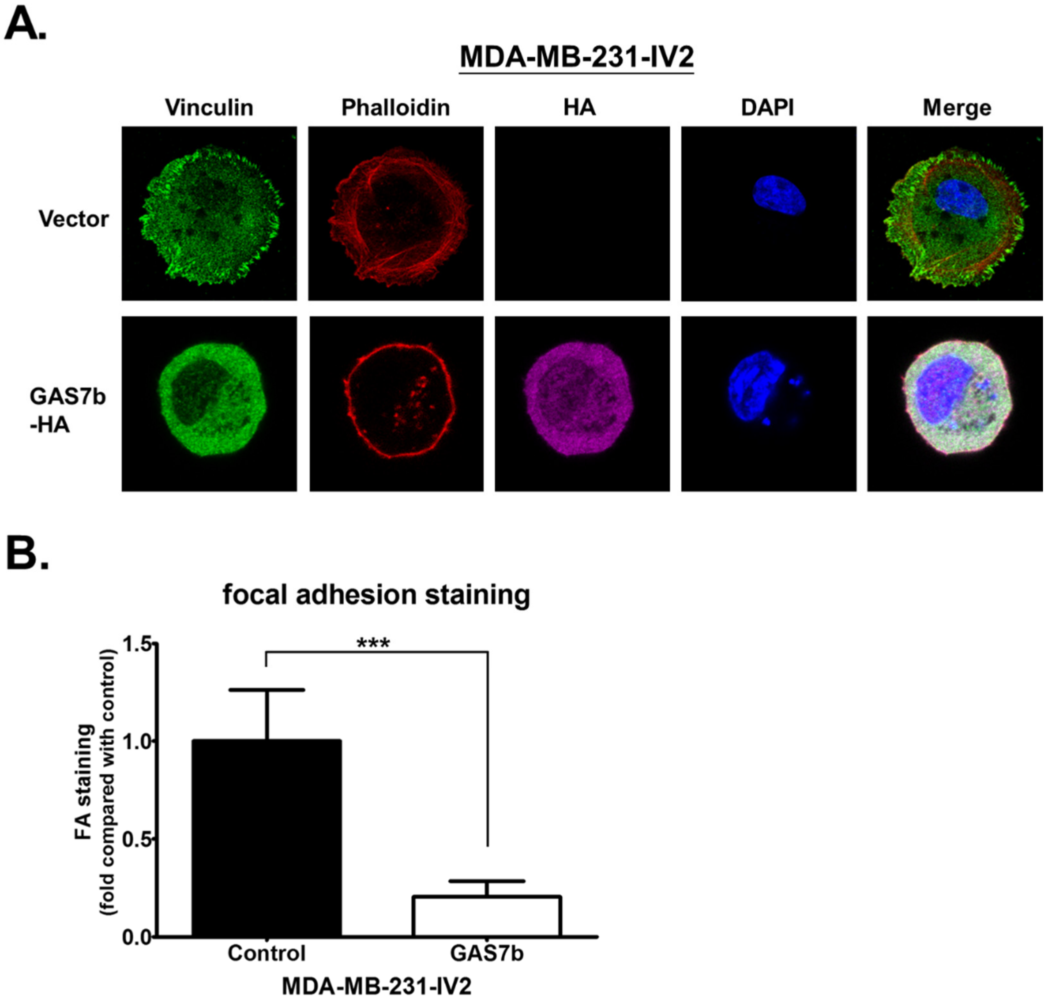

Figure S5. GAS7b disrupts focal adhesions (FAs) formation. MDA-MB-231-IV2 cells were transfected with control, or GAS7b-HA expressing plasmid for 72 hours, and cells were plated on fibronectin coated coverslips for 45 min. (**A**) Immunofluorescence staining and confocal microscopy of Vinculin (green, for FA), Phalloidin (red, for F-actin), HA (purple, for GAS7-HA), and DAPI (blue, for nuclear), (**B**) Quantification for total focal adhesions per cell (n=15, mean±SD). Two-tailed *t*-test was used for statistical analysis (*** $p < 0.001$).

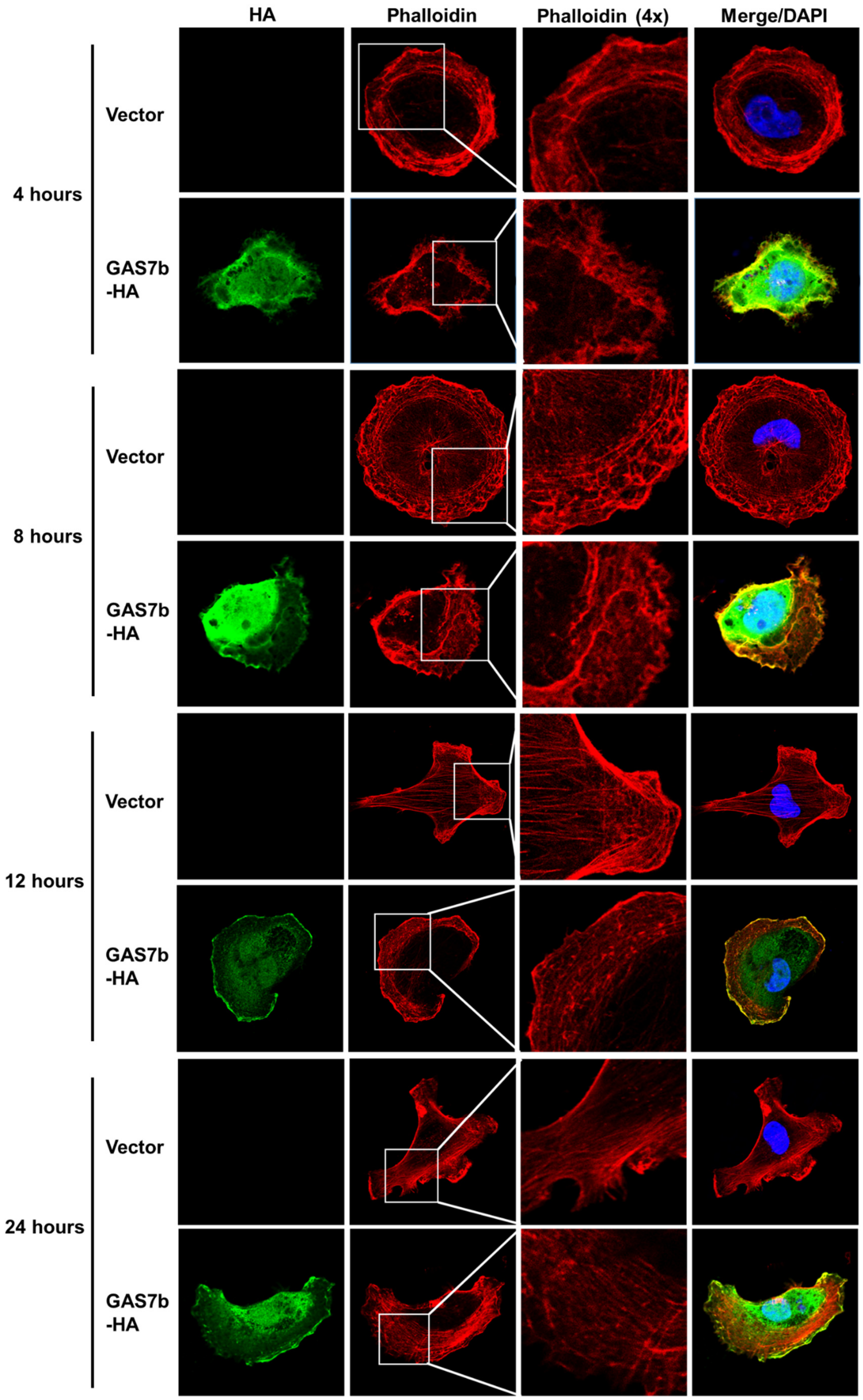
HA
Phalloidin
Phalloidin (4x)
Merge/DAPI
4 hours
Vector
GAS7b
-HA
8 hours
Vector
GAS7b
-HA
12 hours
Vector
GAS7b
-HA
24 hours
Vector
GAS7b
-HA

Figure S6. GAS7b delays actin polymerization to form the actin filaments in MDA-MB-231 cells. MDA-MB-231 cells were transfected with empty vector or GAS7b-HA plasmid for 48 hours, the cells were then plated on FN-coated coverslips for 4, 8, 12, and 24 hours before the fixation of the cells. Immunofluorescence staining of HA (green), Phalloidin (red) and DAPI (blue) was performed, followed by confocal microscopy.

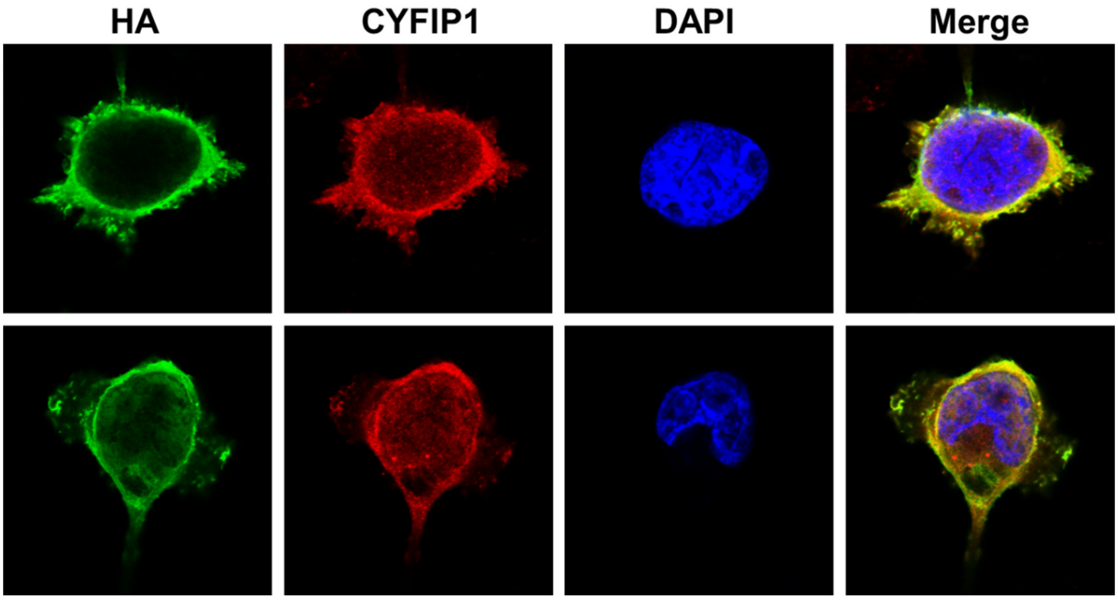

Figure S7. The GAS7b and CYFIP1 protein co-localize at the cell periphery. MDA-MB-231 cells were transfected with GAS7b-HA plasmid for 2 days, the cells were then plated on FN-coated coverslips and immunofluorescence staining for HA (green), CYFIP1 (red) and DAPI (blue) was performed, followed by confocal microscopy.

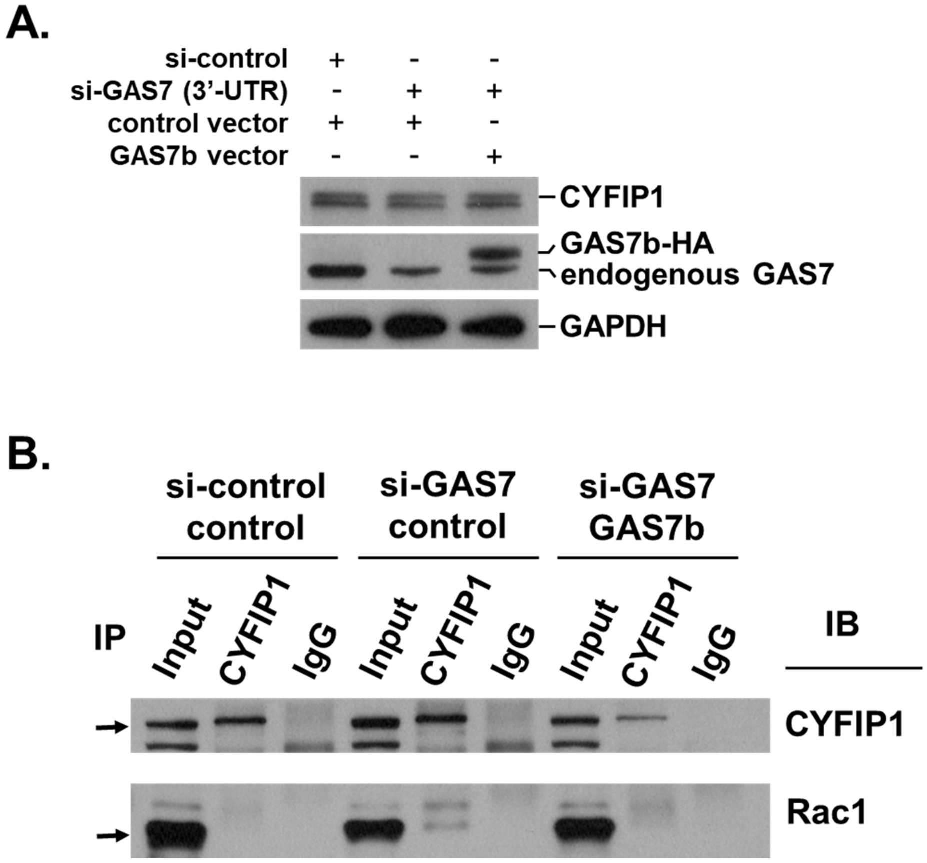

Figure S8. GAS7 protein inhibits CYFIP1 and Rac1 protein-protein interaction. MCF-7 cells were transfected with GAS7 or control siRNA for 12 hours, and these cells were subsequently transfected with GAS7b-HA plasmid for 48 hours. **(A)** The cell lysates were analysis by western blotting assay using anti-CYFIP1, anti-GAS7, and anti-GAPDH antibodies. **(B)** Anti-CYFIP1 antibody was used in the Immunoprecipitation (IP) assay to pulldown associated proteins, and anti-CYFIP1 and anti-Rac1 antibodies were used for Western Blotting. Normal rabbit IgG served as the negative control.

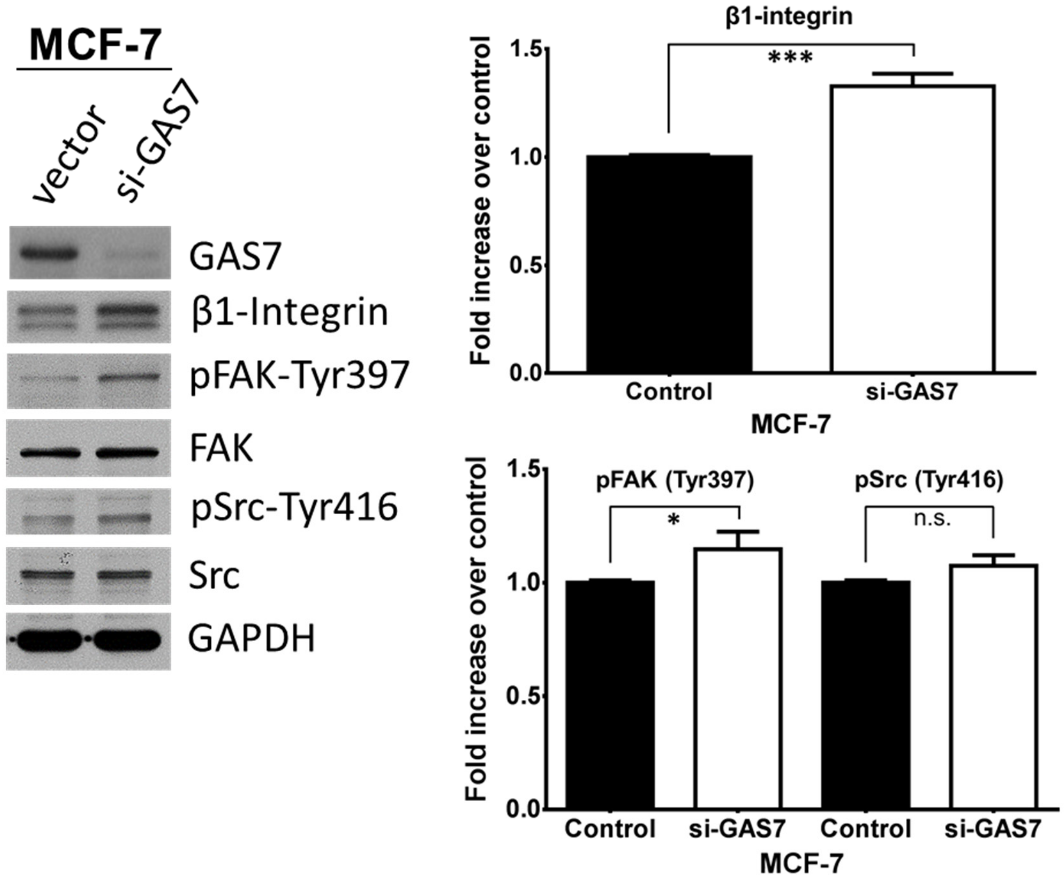

Figure S9. GAS7 suppresses the Integrin/FAK/Src/Rac1 signaling of breast cancer cells. Left: Western Blotting analysis showed β1-Integrin, pFAK (Tyr397), FAK, pSrc (Tyr416), and Src levels in MCF-7 cells with control or GAS7 knockdown. Right: The quantification results of β1-Integrin, pFAK (Tyr397), and pSrc (Tyr416) from Western blotting analysis are shown. Histograms represent normalized mean±SD (n=3). Two-tailed *t*-test was used for the statistical analysis (* $p < 0.05$; *** $p < 0.001$; n.s., non-significant).

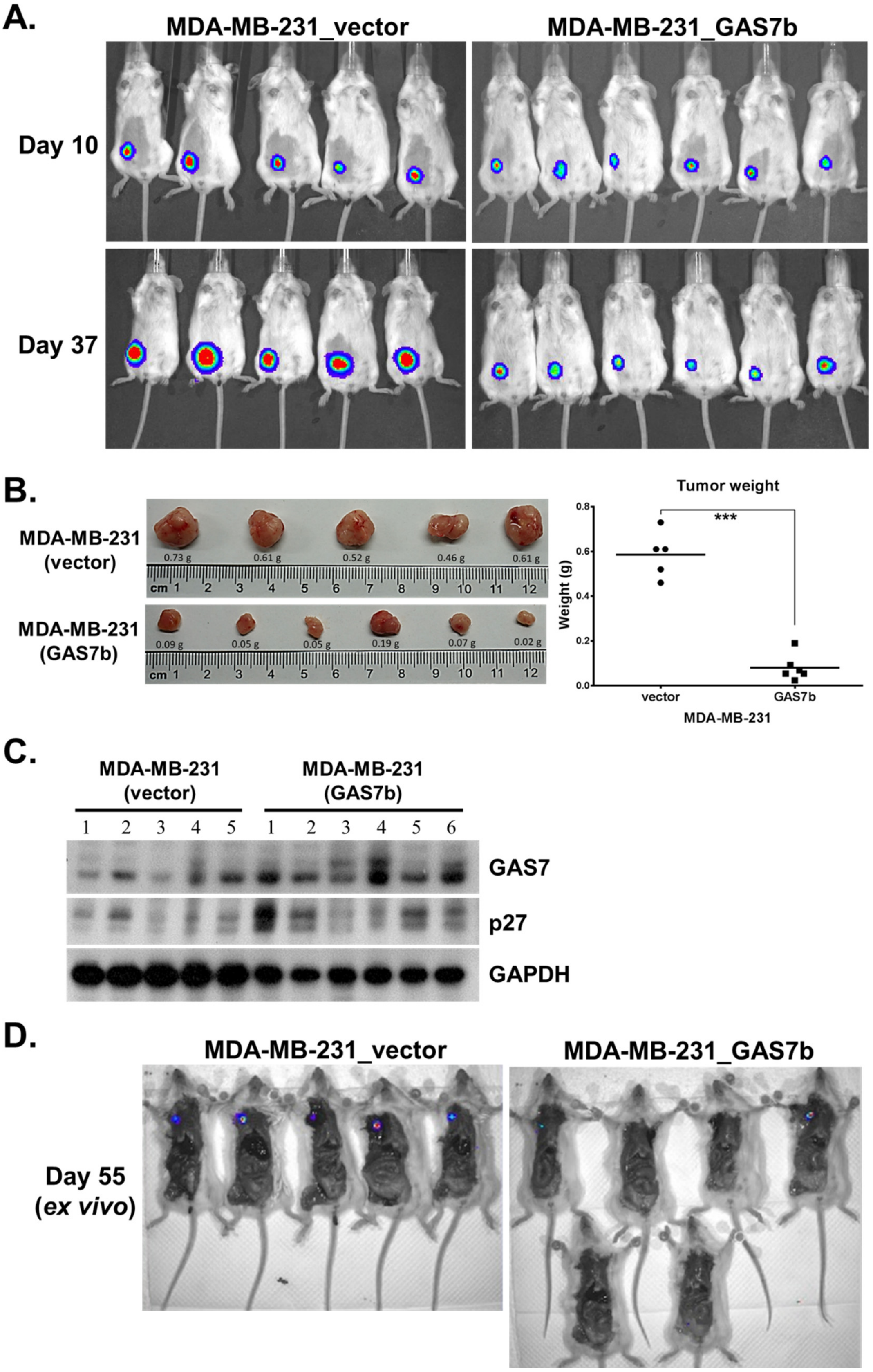

A.
MDA-MB-231_vector
MDA-MB-231_GAS7b
Day 10
Day 37
B.
MDA-MB-231 (vector)
MDA-MB-231 (GAS7b)
0.73 g
0.61 g
0.52 g
0.46 g
0.61 g
0.09 g
0.05 g
0.05 g
0.19 g
0.07 g
0.02 g
Tumor weight
Weight (g)
***
vector
GAS7b
MDA-MB-231
C.
MDA-MB-231 (vector)
MDA-MB-231 (GAS7b)
1 2 3 4 5
1 2 3 4 5 6
GAS7
p27
GAPDH
D.
MDA-MB-231_vector
MDA-MB-231_GAS7b
Day 55 (ex vivo)

Figure S10. GAS7b reduces MDA-MB-231 breast cancer cells proliferation and metastasis in the orthotopic mouse model. (A) The tumors were monitored by BLI technologies on day 10 and day 37 after implantation of 10^6 cells orthotopically into mouse mammary fat pads. **(B)** The mice were sacrificed on day 55, primary tumor size (left) and tumor weight (right) were measured. Two-tailed t-test was used for statistical analysis (*** $p < 0.001$). **(C)** The orthotopically implanted primary tumors were analyzed for GAS7 and p27 expression by Western blotting. **(D)** The *ex-vivo* BLI analysis was used to detect axillary lymph-node metastasis after sacrifice of the mice on day 55. The primary tumors were removed before photography.

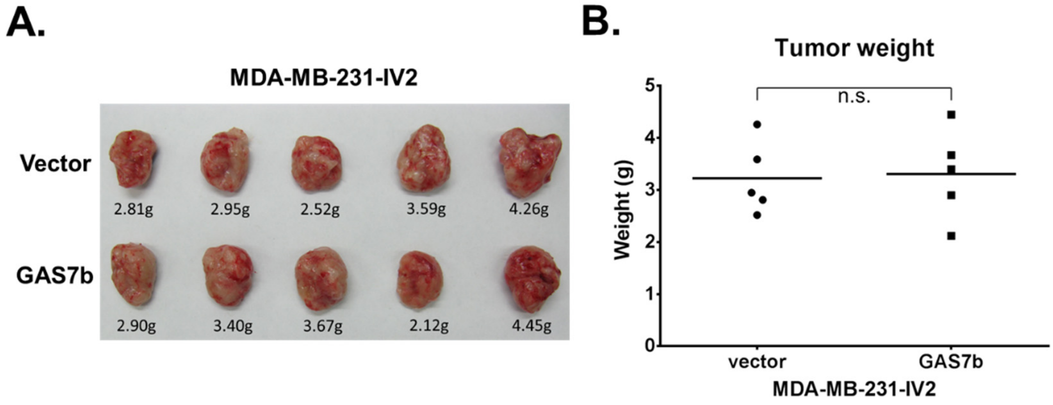

Figure S11. GAS7b did not reduces MDA-MB-231-IV2 breast cancer cells growth ability in orthotopic mouse model. $1x10^6$ MDA-MB-231-IV2 cells stably expressing GAS7b or control vector were orthotopically implanted into mammary fat pads of female SCID mice. The mice were sacrificed on day 56, primary tumor size **(A)** and tumor weight **(B)** were measured. Two-tailed t-test was used for these statistical analysis (n.s., non-significant).

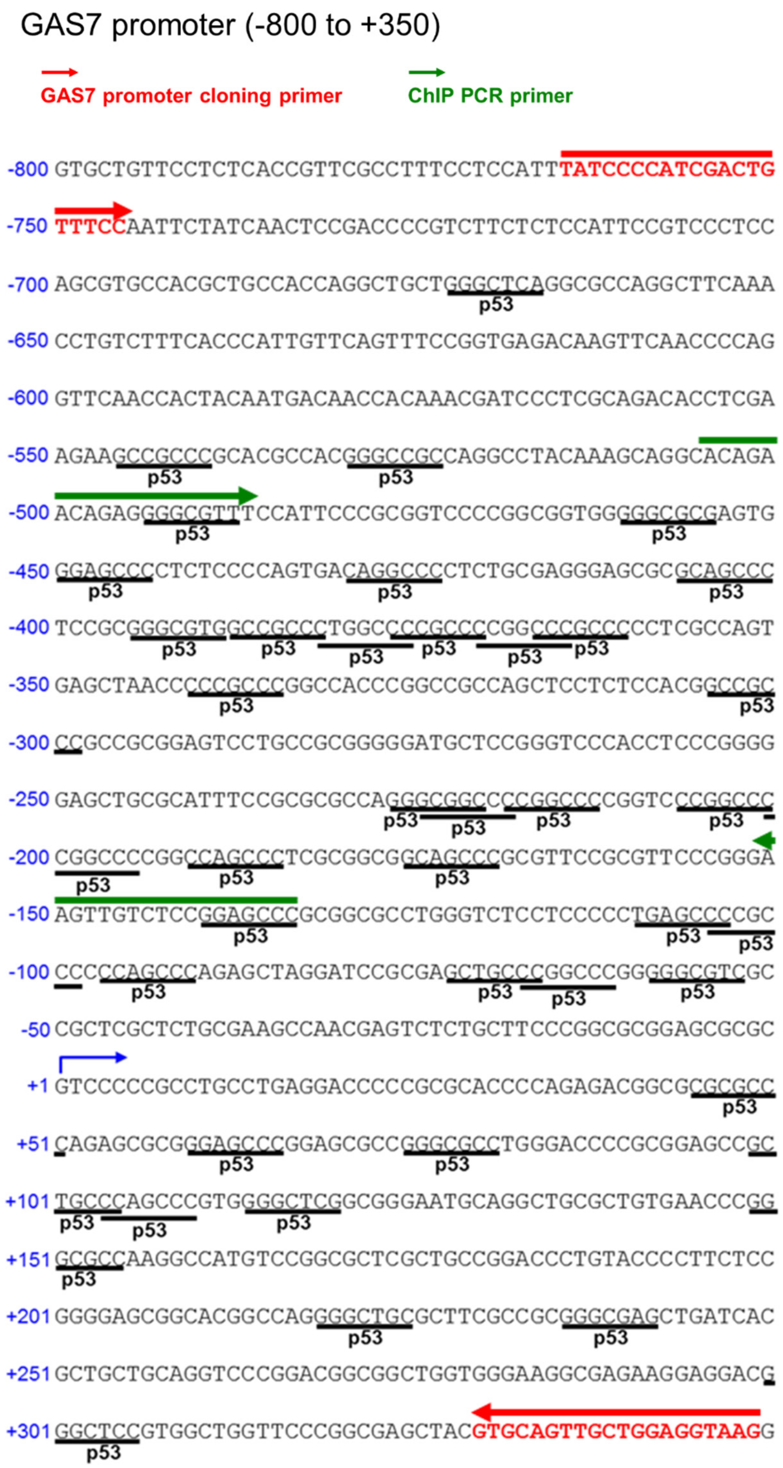

Figure S12. GAS7 promoter, exon1 sequences and putative p53 binding sites. PROMO website (http://alggen.lsi.upc.es/) was used to identify the p53 binding sites in GAS7 promoter and exon1 region (-800 bp to +350 bp). Black lines show the putative binding site of p53, red arrows define the primer regions for promoter cloning for reporter plasmid construct and activity assay, and green arrows are the ChIP-PCR primer sites.

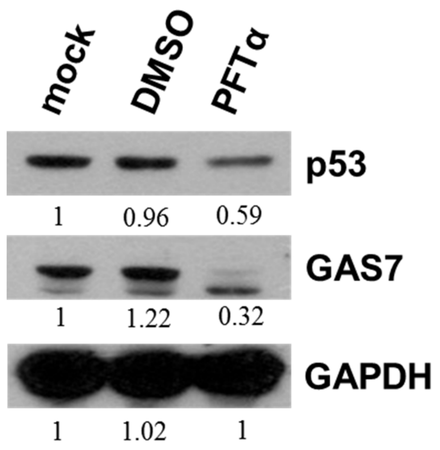

Figure S13. The p53 inhibitor downregulates GAS7 expression in MCF-7 cells. MCF-7 cells were treated for 72 hours with DMSO or 30 μM of Pifithrin-α (PFTα), a p53 inhibitor, and the protein lysates were analyzed by Western blotting using anti-p53, anti-GAS7 and anti-GAPDH antibodies. GAPDH was served as the loading control.

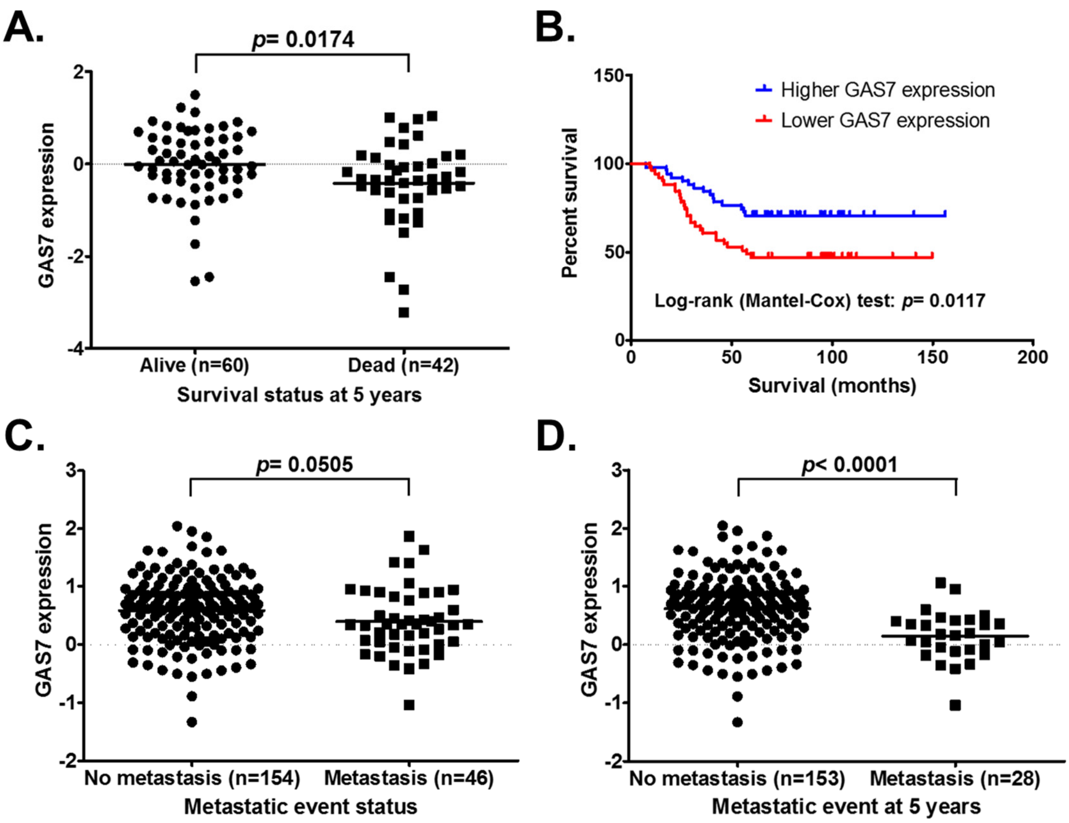

Figure S14. Lower GAS7 expression level is correlated with poor prognosis of breast cancer. Several public domain of breast cancer gene expression array datasets were compiled from Oncomine database. The GAS7 gene expression level was extracted from these datasets and correlated with survival and metastatic event of the patients. The Bild breast cancer dataset (GSE3143) was analyzed for **(A)** the relationship between GAS7 mRNA expression level and 5 years survival of patients, and **(B)** the Kaplan-Meier analyses of overall survival between GAS7-high and GAS7-low expression groups. Log-rank test for trend was used for statistical analysis. The Schmidt breast cancer dataset (GSE11121) was analyzed for the correlation of GAS7 mRNA expression and metastatic event **(C)**, or metastatic event within 5 years **(D)**. Two-tailed t-test was used for these statistical analysis.

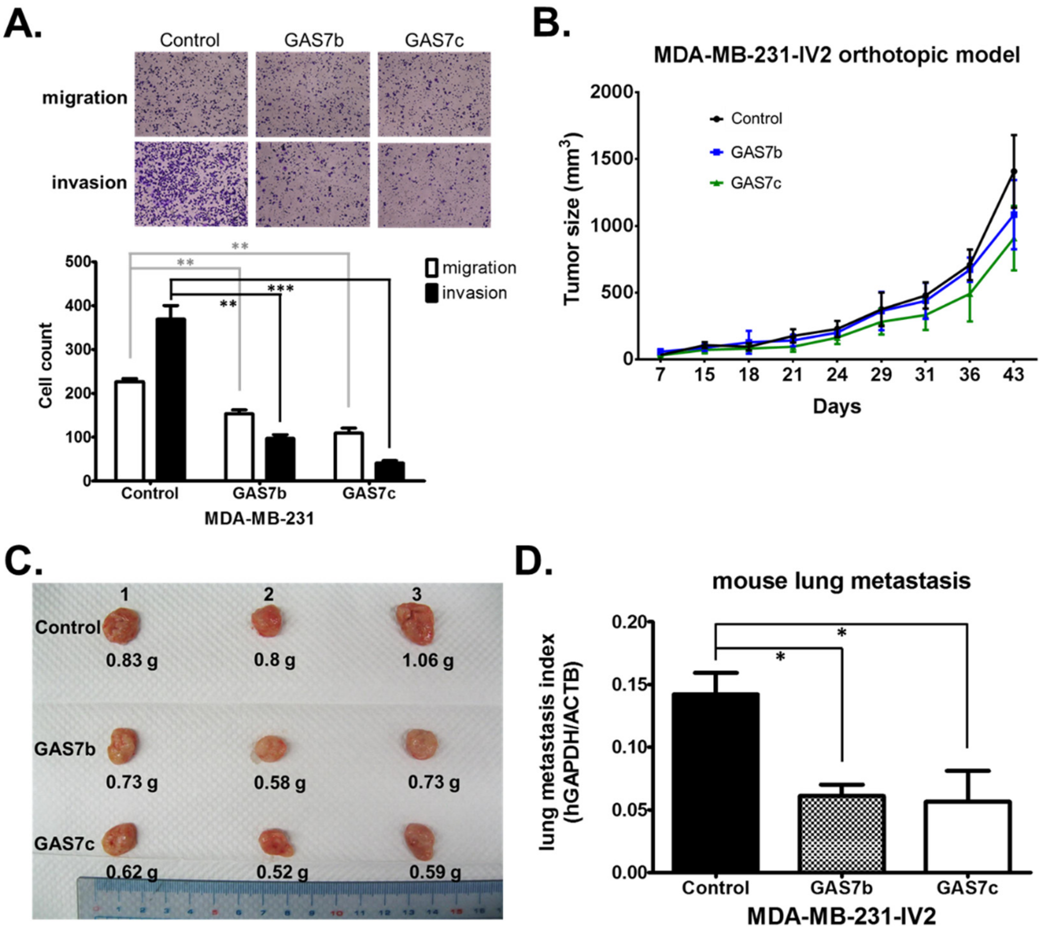

Figure S15. GAS7b and GAS7c reduced breast cancer cells proliferation and metastasis ability in cell based and mouse models. MDA-MB-231 cells overexpressing empty control, GAS7b or GAS7c plasmid. **(A)** Upper panels: representative photographs from trans-well cell migration and invasion assays. Lower panel: statistical analysis of migrated and invaded cells are shown by histograms. Data represents normalized mean±SD (n=3), and Two-tailed *t*-test was used for statistical analysis (** $p< 0.01$; *** $p< 0.001$). The MDA-MB-231-IV2 cells stably expressing control, GAS7b or GAS7c in mammary fat pad orthotopic mouse model. The tumor growth curves are shown in **(B).** The statistical significance was calculated using linear regression model (GAS7b vs. Control, p=0.162; Gas7c vs. Control, p=0.0018). **(C)** The tumor size and weight are shown from tumors harvested on day 43 after implantation in orthotopic mouse model. **(D)** qRT-PCR assay was perform to measure the lung metastasis of MDA-MB-231-IV2 cells. Lung metastasis index was calculated by human-specific GAPDH levels normalized by total actin from mouse lung. Histograms represent normalized mean±SD (n=3). Student's *t*-test was used for statistical analysis (* $p< 0.05$).

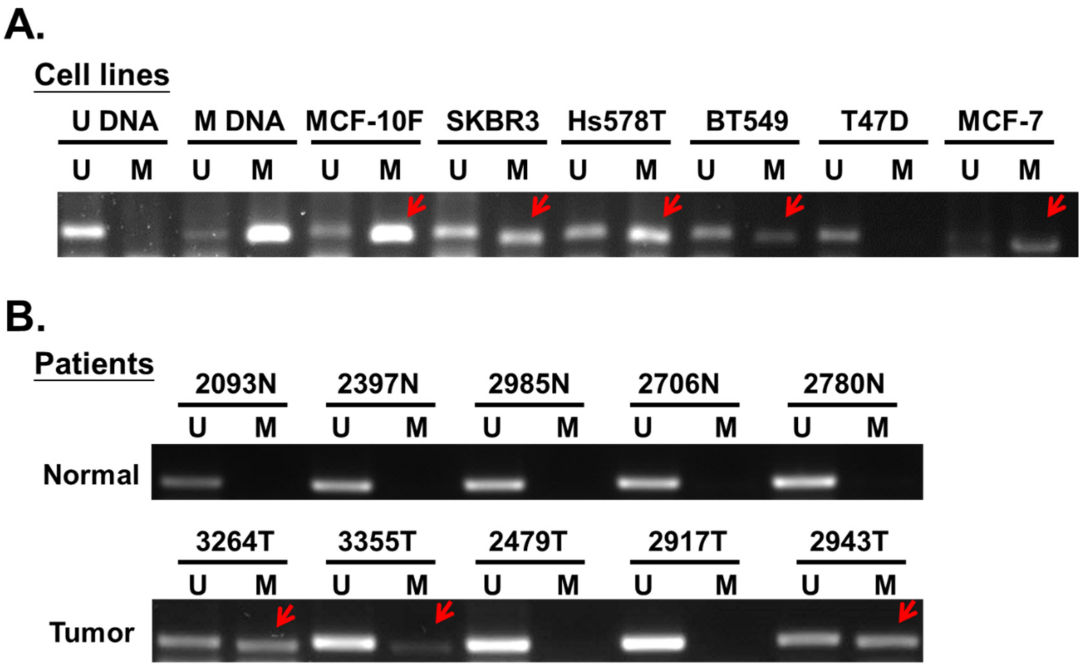

Figure S16. GAS7 gene expression is likely regulated by DNA methylation. The methylation-specific PCR (MSP) was performed to detect promoter methylation of the GAS7 gene. U primer and M primer were used for detecting the unmethylated and methylated DNA, respectively. U DNA and M DNA were used as positive controls of U and M PCR reactions. **(A)** Various breast cancer cell lines, and **(B)** paired breast normal (N) and tumor samples (T) were analyzed by MSP. Red arrows indicate the sample having promoter methylation on GAS7 gene.

Supplemental Tables

Table S1: Primer sequences in PCR reaction.

	**Primer name**	**Sequence (5' to 3')**
**qRT-PCR**		
GAS7(Total form)	GAS7-F	AGACACGATGCCGGAACAG
	GAS7-R	TTATCCTTTCCCGGATGAATTCT
beta-actin	ACTB-F	CGGCATCGTCACCAACTG
	ACTB-R	TCTCAAACATGATCTGGGTCATCT
GAS7a	GAS7a-F	CTTTGACGATGTTTCTTG
	GAS7a-R	GTTTATTGTGATGGTGTTT
GAS7b	GAS7b-F	GCAGGACCCAAGACTCTC
	GAS7b-R	ACCATTCCAGGCTTCATCA
GAS7c	GAS7c-F	CTACGTGCAGTTGCTGGA
	GAS7c-R	GGAAGGATGACCGTCTGG
GAS7d	GAS7d-F	CTAAAGCGAGTGTCTGAC
	GAS7d-R	CATTCCAGGCTTCTTATCC
GAPDH(Human)	hGAPDH-F	TCCTGGTATGACAACGAAT
	hGAPDH-R	GGTCTCTCTCTTCCTCTTG
**Gene cloning**		
GAS7b (pcDNA3.1)	Gas7b-CDS-F(Xho I)	ATCGctcgagAGCTGTTCTCTGATGAAGCC#
	Gas7b-CDS-R(Afl II)	ATCGcttaagTTCTGGAATCACCAGCTCTC#
GAS7b-HA (pCMV-HA)	Gas7b-HA-CDS(SalI)-F	AATgtcgacAATGAAGCCTGGAATGGTCC#
	Gas7b-HA-CDS(NotI)-R	ATATgcggccgcTTCTGGAATCACCAGCTCTC#
GAS7 promoter (pGL4.20)	Gas7-Promoter-F(NheI)	AAgctagcAAAAGCATTGGTGCTGTTCC#
	Gas7-Promoter-R(HindIII)	AAaagcttAGCAGAGACTCGTTGGCTTC#
GAS7c (pJET1.2)	Gas7c(CDS)-F	GCAGGCTGCGCTGTGAAC
	Gas7c(CDS)-R	GAAGCTGCACAGGCCCATCT
**ChIP-PCR**		
GAS7(ChIP)	GAS7(ChIP)-F	ACAGAACAGAGGGGCGTTT
	GAS7(ChIP)-R	GGGCTCCGGAGACAACTT
**Site directed mutagenesis**		
P53(R175H)	p53-524GA-F	ATGACGGAGGTTGTGAGGCACTGCCCCCACCAT

		GA
	p53-524GA-R	TCATGGTGGGGGCAGTGCCTCACAACCTCCGTCAT
P53(G244D)	p53-731GA-F	CAGTTCCTGCATGGACGGCATGAACCGGAG
	p53-731GA-R	CTCCGGTTCATGCCGTCCATGCAGGAACTG
P53(R248Q)	p53-743GA-F	ATGGGCGGCATGAACCAGAGGCCCATCCTCACCAT
	p53-743GA-R	ATGGTGAGGATGGGCCTCTGGTTCATGCCGCCCAT
P53(R342X)	p53-1024CT-F	CGCTTCGAGATGTTCTGAGAGCTGAATGAG
	p53-1024CT-R	CTCATTCAGCTCTCAGAACATCTCGAAGCG
**Methylation-specific PCR (MSP)**		
GAS7 methylation	GAS7-M-F	AGTTTCGTTTTTTAGTTTAGAGTTAGGATTCGC
	GAS7-M-R	GCGAAAATCCTCAAACAAACGAAAAACG
GAS7 unmethylation	GAS7-U-F	GAGTTTTGTTTTTTAGTTTAGAGTTAGGATTTGTG
	GAS7-U-R	AAATACACAAAAATCCTCAAACAAACAAAAAACA
**Gene knockdown**		
GAS7-siRNA-1	GAS7-homo-316	GGAGCUACUGCGACUACUUtt
GAS7-siRNA-2	GAS7-homo-899	CCCAGUCCAAAUGGUUUGAtt
GAS7-siRNA-3	GAS7-homo-1009	CGGCAUGAAACAGACAUGUtt
GAS7-siRNA (3'-UTR)	Hs.Ri.GAS7.13.1	CUUGAAACAUUGCAACCAAAAAGGT
Control siRNA	Negative control	UUCUCCGAACGUGUCACGUTT

the restriction enzyme digestion site is shown in lower case letters.

Table S2: Sequenom MassArray assay for TP53 mutation (110 mutations).

No.	Mutation position	Event	Amino Acid Change	No.	Mutation position	Event	Amino Acid Change
1	193	A>T	R65Stop	56	713	G>A	C238Y
2	305	C>T	T102I	57	713	G>T	C238F
3	359	A>T	K120M	58	715	A>G	N239D
4	392	A>T	N131I	59	722	C>T	S241F
5	395	A>G	K132R	60	725	G>T	C242F
6	404	G>A	C135Y	61	725	G>A	C242Y
7	404	G>T	C135F	62	730	G>A	G244S
8	406	C>T	Q136Stop	63	730	G>T	G244C
9	413	C>T	A138V	64	731	G>A	G244D
10	422	G>A	C141Y	65	733	G>A	G245S
11	430	C>T	Q144Stop	66	733	G>T	G245C
12	437	G>A	W146Stop	67	734	G>A	G245D
13	438	G>A	W146Stop	68	734	G>T	G245V
14	451	C>T	P151S	69	736	A>G	M246V
15	455	C>T	P152L	70	742	C>T	R248W
16	461	G>T	G154V	71	743	G>A	R248Q
17	469	G>T	V157F	72	743	G>T	R248L
18	473	G>A	R158H	73	745	A>G	R249G
19	473	G>T	R158L	74	746	G>T	R249M
20	475	G>C	A159P	75	747	G>T	R249S
21	476	C>T	A159V	76	748	C>A	P250T
22	481	G>A	A161T	77	749	C>T	P250L
23	484	A>T	I162F	78	752	T>C	I251T
24	488	A>G	Y163C	79	763	A>T	I255F
25	493	C>T	Q165Stop	80	772	G>A	E258K
26	496	T>A	S166T	81	796	G>A	G266R
27	499	C>T	Q167Stop	82	797	G>A	G266E
28	517	G>A	V173M	83	797	G>T	G266V
29	517	G>T	V173L	84	814	G>A	V272M
30	524	G>A	R175H	85	814	G>T	V272L
31	524	G>T	R175L	86	817	C>T	R273C
32	527	G>T	C176F	87	818	G>A	R273H
33	527	G>A	C176Y	88	818	G>T	R273L
34	535	C>T	H179Y	89	818	G>C	R273P
35	536	A>G	H179R	90	824	G>A	C275Y
36	536	A>T	H179L	91	824	G>T	C275F
37	569	C>T	P190L	92	830	G>T	C277F
38	574	C>T	Q192Stop	93	832	C>T	P278S
39	577	C>T	H193Y	94	832	C>A	P278T
40	578	A>G	H193R	95	833	C>T	P278L
41	578	A>T	H193L	96	836	G>A	G279E
42	581	T>G	L194R	97	838	A>G	R280G
43	584	T>C	I195T	98	839	G>C	R280T
44	586	C>T	R196Stop	99	839	G>A	R280K
45	610	G>T	E204Stop	100	841	G>C	D281H
46	614	A>G	Y205C	101	844	C>T	R282W
47	637	C>T	R213Stop	102	844	C>G	R282G
48	641	A>G	H214R	103	853	G>A	E285K
49	646	G>A	V216M	104	856	G>A	E286K
50	659	A>G	Y220C	105	856	G>C	E286Q
51	667	C>A	P223T	106	877	G>C	G293R
52	701	A>G	Y234C	107	880	G>T	E294Stop
53	707	A>G	Y236C	108	892	G>T	E298Stop
54	711	G>A	M237I	109	916	C>T	R306Stop
55	711	G>T	M237I	110	1024	C>T	R342Stop

Table S3. TP53 gene mutation sites in 339 Taiwanese breast cancer patients

Nucleotide changes	AA Number	WT AA	Mut AA	no. of cases (n=339)	protein domain
152C>T	51	S	L	1	
177_178 ins CTCC	59	P	fs	1	
178C>T	60	Q	Stop	1	
178 del.C	60	Q	fs	1	
245A>G	82	H	R	1	
246_247 del	82_83del			1	
263A>G	88	Y	C	2	
299T>G	100	I	S	1	
311A>G	104	Y	C	1	
315G>A	105	M	I	1	
317G>A	106	C	Y	1	
326C>A	109	S	Y	1	DNA-binding domain
329G>A	110	C	Y	1	DNA-binding domain
332T>C	111	L	P	1	DNA-binding domain
337G>T	113	G	C	2	DNA-binding domain
359A>T	120	K	M	1	DNA-binding domain
375_376 ins TGCAC	125	T	fs	1	DNA-binding domain
375G>A	125	T	T	1	DNA-binding domain
406C>T	136	Q	Stop	1	DNA-binding domain
415 del.G	139	E	fs	1	DNA-binding domain
437G>A	146	W	Stop	1	DNA-binding domain
451C>T	151	P	S	1	DNA-binding domain
453 insert	151	R	delinsRDRR	1	DNA-binding domain
457G>A	153	E	K	1	DNA-binding domain
469G>T	157	V	F	1	DNA-binding domain
475G>C	159	A	P	1	DNA-binding domain
481G>A	161	A	T	1	DNA-binding domain
488A>G	163	Y	C	1	DNA-binding domain
517G>A	173	V	M	1	DNA-binding domain
520C>T	174	R	Stop	2	DNA-binding domain
524G>A	175	R	H	4	DNA-binding domain
527G>A	176	C	Y	1	DNA-binding domain
536A>G	179	H	R	1	DNA-binding domain
569C>T	190	P	L	1	DNA-binding domain
574C>T	192	Q	Stop	2	DNA-binding domain

578A>G	193	H	R	1	DNA-binding domain
581T>G	194	L	R	1	DNA-binding domain
586C>T	196	R	Stop	1	DNA-binding domain
610G>T	204	E	Stop	1	DNA-binding domain
637C>T	213	R	Stop	1	DNA-binding domain
641A>G	214	H	R	3	DNA-binding domain
659A>G	220	Y	C	1	DNA-binding domain
711G>A	237	M	I	1	DNA-binding domain
713G>T	238	C	F	1	DNA-binding domain
722C>T	241	S	F	2	DNA-binding domain
725G>T	242	C	F	1	DNA-binding domain
731G>A	244	G	D	3	DNA-binding domain
733G>A	245	G	S	2	DNA-binding domain
733G>A	245	G	V	1	DNA-binding domain
736T>C	246	S	P	1	DNA-binding domain
743G>A	248	R	W	1	DNA-binding domain
743G>A	248	R	Q	5	DNA-binding domain
814G>A	272	V	M	1	DNA-binding domain
818G>A	273	R	H	2	DNA-binding domain
833C>T	278	P	L	1	DNA-binding domain
844C>T	282	R	W	1	DNA-binding domain
856G>A	286	E	K	1	DNA-binding domain
892G>T	298	E	Stop	1	
916C>T	306	R	Stop	1	
1024C>T	342	R	Stop	4	tetramerization motif

Supplemental References

1 Rhodes DR, Yu J, Shanker K, Deshpande N, Varambally R, Ghosh D *et al*. ONCOMINE: a cancer microarray database and integrated data-mining platform. Neoplasia 2004; 6: 1-6.

2 Curtis C, Shah SP, Chin SF, Turashvili G, Rueda OM, Dunning MJ *et al*. The genomic and transcriptomic architecture of 2,000 breast tumours reveals novel subgroups. Nature 2012; 486: 346-352.

3 Bild AH, Yao G, Chang JT, Wang Q, Potti A, Chasse D *et al*. Oncogenic pathway signatures in human cancers as a guide to targeted therapies. Nature 2006; 439: 353-357.

4 Schmidt M, Bohm D, von Torne C, Steiner E, Puhl A, Pilch H *et al*. The humoral immune system has a key prognostic impact in node-negative breast cancer. Cancer research 2008; 68: 5405-5413.

5 Pawitan Y, Bjohle J, Amler L, Borg AL, Egyhazi S, Hall P *et al*. Gene expression profiling spares early breast cancer patients from adjuvant therapy: derived and validated in two population-based cohorts. Breast cancer research : BCR 2005; 7: R953-964.

6 Chan SH, Huang WC, Chang JW, Chang KJ, Kuo WH, Wang MY *et al*. MicroRNA-149 targets GIT1 to suppress integrin signaling and breast cancer metastasis. Oncogene 2014; 33: 4496-4507.

7 Lin KT, Yeh YM, Chuang CM, Yang SY, Chang JW, Sun SP *et al*. Glucocorticoids mediate induction of microRNA-708 to suppress ovarian cancer metastasis through targeting Rap1B. Nat Commun 2015; 6: 5917.
